# Novel Plastid Genome Characteristics in *Fugacium kawagutii* and the Trend of Accelerated Evolution of Plastid Proteins in Dinoflagellates

**DOI:** 10.1093/gbe/evad237

**Published:** 2023-12-29

**Authors:** Jiamin He, Yulin Huang, Ling Li, Sitong Lin, Minglei Ma, Yujie Wang, Senjie Lin

**Affiliations:** State Key Laboratory of Marine Environmental Science, College of Ocean and Earth Sciences, Xiamen University, Xiamen 361102, China; State Key Laboratory of Marine Environmental Science, College of Ocean and Earth Sciences, Xiamen University, Xiamen 361102, China; State Key Laboratory of Marine Environmental Science, College of Ocean and Earth Sciences, Xiamen University, Xiamen 361102, China; State Key Laboratory of Marine Environmental Science, College of Ocean and Earth Sciences, Xiamen University, Xiamen 361102, China; State Key Laboratory of Marine Environmental Science, College of Ocean and Earth Sciences, Xiamen University, Xiamen 361102, China; State Key Laboratory of Marine Environmental Science, College of Ocean and Earth Sciences, Xiamen University, Xiamen 361102, China; State Key Laboratory of Marine Environmental Science, College of Ocean and Earth Sciences, Xiamen University, Xiamen 361102, China; Department of Marine Sciences, University of Connecticut, Groton, CT 06340, USA

**Keywords:** dinoflagellates, minicircles, plastid, symbiodiniaceae, core regions, codon usage bias

## Abstract

Typical (peridinin-containing) dinoflagellates possess plastid genomes composed of small plasmids named “minicircles”. Despite the ecological importance of dinoflagellate photosynthesis in corals and marine ecosystems, the structural characteristics, replication dynamics, and evolutionary forcing of dinoflagellate plastid genomes remain poorly understood. Here, we sequenced the plastid genome of the symbiodiniacean species *Fugacium kawagutii* and conducted comparative analyses. We identified *psbT*-coding minicircles, features previously not found in Symbiodiniaceae. The copy number of *F. kawagutii* minicircles showed a strong diel dynamics, changing between 3.89 and 34.3 copies/cell and peaking in mid-light period. We found that *F. kawagutii* minicircles are the shortest among all dinoflagellates examined to date. Besides, the core regions of the minicircles are highly conserved within genus in Symbiodiniaceae. Furthermore, the codon usage bias of the plastid genomes in Heterocapsaceae, Amphidiniaceae, and Prorocentraceae species are greatly influenced by selection pressure, and in Pyrocystaceae, Symbiodiniaceae, Peridiniaceae, and Ceratiaceae species are influenced by both natural selection pressure and mutation pressure, indicating a family-level distinction in codon usage evolution in dinoflagellates. Phylogenetic analysis using 12 plastid-encoded proteins and five nucleus-encoded plastid proteins revealed accelerated evolution trend of both plastid- and nucleus-encoded plastid proteins in peridinin- and fucoxanthin-dinoflagellate plastids compared to plastid proteins of nondinoflagellate algae. These findings shed new light on the structure and evolution of plastid genomes in dinoflagellates, which will facilitate further studies on the evolutionary forcing and function of the diverse dinoflagellate plastids. The accelerated evolution documented here suggests plastid-encoded sequences are potentially useful for resolving closely related dinoflagellates.

**Significance**

Despite the importance of dinoflagellates in coral reef and general marine ecosystems, our structural and functional understanding of their unusual plastids is very limited. Here, we isolated and molecularly dissected the plastid genome of a coral-associated (symbiodiniacean) species. We documented its minicircular chromosomes, one of which was thought absent in Symbiodiniaceae, and shed light on structural conservation and innovation. Furthermore, we observed a strong diel dynamic of minicircle replication, codon usage bias of minicircles and revealed accelerated evolution of plastid proteins in peridinin dinoflagellate. Our results provide novel insights into dinoflagellate plastid genome structural and functional evolution with implications in using plastid protein genes to phylogenetically resolve closely related Symbiodiniaceae and dinoflagellate lineages.

## Introduction

Dinoflagellates are globally distributed in both freshwater and marine environments. They are significant primary producers, only second to diatoms in productivity (Field et al. 1998). Dinoflagellates are an extremely diverse group of protists with photosynthetic species and nonphotosynthetic species (Liu 2015). Most photosynthetic dinoflagellates are mixotrophic (Jeong et al. 2010; Hansen 2011), switching between feeding and photosynthesis depending on environmental nutrient conditions, which enhance growth rate and photosynthetic efficiency (Li et al. 1999). Dinoflagellate plastids are very complex. Typical dinoflagellate plastids arise from secondary endosymbiosis of rhodophyte origin, which contain peridinin as the major accessory pigment. Some species have lost the ancestral plastid and acquired a plastid of chlorophyte origin, a process named serial secondary endosymbiosis. Others of the species that had lost the original chloroplast gained a plastid of haptophyte or diatom (secondary plastids themselves) origin, which is considered tertiary replacement plastid (Keeling 2010). Most plastid genomes in Plantae are encoded on a single chromosome and contain 90 to 250 genes (Dorrell and Howe 2015), but the peridinin dinoflagellate plastid genome has been fragmented into a population of minicircles containing none to only a few genes each, commonly called “minicircles” (Zhang et al. 1999; Howe et al. 2008). As most of the plastid genes have been transferred to the nuclear genome, less than 20 genes in total (16 to 17 protein-coding plus nonprotein coding genes) are retained in plastid genomes. The sizes of minicircles are 2 to 5 kbp, and each minicircle contains a core region. The function of the core region is unclear, but it contains direct and inverted repeats and is thought to promote the polycistronic transcription of the circles (Moore et al. 2003; Waller and Kořený 2017).

The minicircle plastid genomes have only been characterized for a handful of species, including *Heterocapsa triquetra*, *Amphidinium carterae*, *A. operculatum*, *Tripos horridus,* and *Symbiodinium* sp*. Clade C3* (potentially *Cladocopium sodalum* following the recent revision by Butler et al. [2023]) (Zhang et al. 1999; Barbrook and Howe 2000; Barbrook et al. 2001; Hiller 2001; Koumandou et al. 2004; Zauner et al. 2004; Barbrook et al. 2006; Barbrook et al. 2014; Liu 2015). It has been shown that dinoflagellate plastids are changed under selection pressure throughout their evolution, commencing after their divergence from other plastid lineages, and throughout the radiation of extant species (Dorrell et al. 2017). Codon usage bias analysis is a suitable strategy for identifying the principal evolutionary driving forces in different organisms (Gao et al. 2022), because codon usage is shaped by the balance between mutation and natural selection (Duret 2002; Hershberg and Petrov 2008). While mutations are random processes that can occur spontaneously, natural selection is a nonrandom process that involves the survival and reproduction of individuals with advantageous traits in their environment (Barton 2010). However, there is little information on codon usage bias in dinoflagellates. More generally, research about peridinin dinoflagellate plastid genomes is still limited, and new insights can arise from more systematic and comparative analyses as well as investigation of copy number differences among minicircles and replication dynamics of the minicircles.

In this study, we isolated the plastid of *Fugacium kawagutii* and sequenced its genome. We also quantified minicircle copy number and investigated its dynamics in the light/dark cycle. In addition, we integrated the existing dinoflagellate plastid genome sequence data and conducted comparative analysis as well as phylogenetic analysis based on the data. The study was aimed to address three major questions: (i) What are the major structural and duplication characteristics of *F. kawagutii* minicircles? (ii) How did the plastid genome evolve in peridinin dinoflagellates in general and in Symbiodiniaceae in particular? (iii) How did the nucleus- as well as plastid-encoded plastid proteins of dinoflagellates evolve in comparison to the plastid proteins of nondinoflagellate algae?

## Results

### The Sequencing and Characterization of *F. kawagutii* Minicircle Plastid Genome

**Fig. 1.**
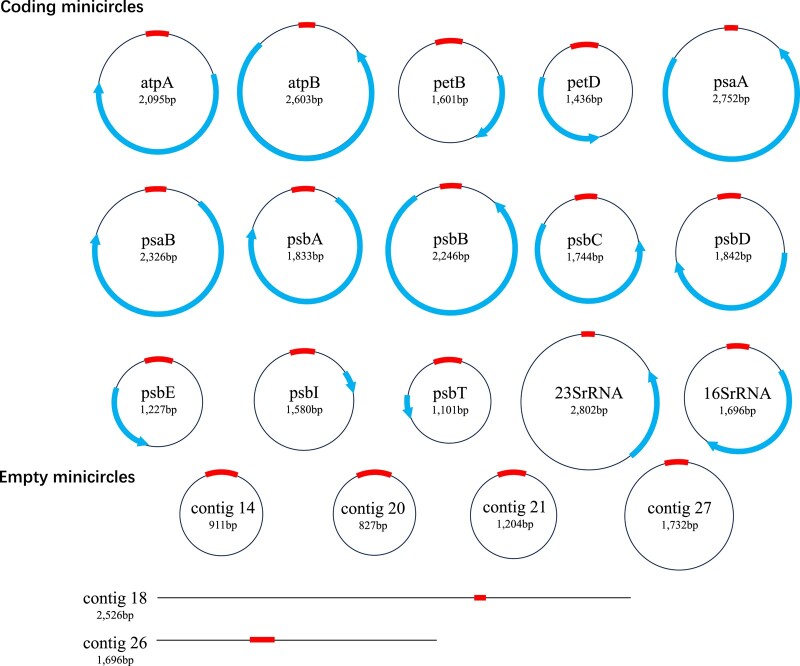
The *Fugacium kawagutii* chloroplast genome. The core region of each minicircle is shown by a thick arch positioned on the top of the circle; coding region is shown by a thick curved arrow. Contig18 and contig 26 could not form circle based on our data, both contain the core region (thick line).

We obtained a relatively clean plastid preparation and sequenced the plastid genome using short-read high-throughput Illumina technology. PCR amplification and sequencing of the amplicons were also carried out when necessary to obtain the full length. The 30 contigs (505 to 2,802 bp) resulting from the Illumina sequencing were assembled into 19 minicircles (Fig. 1, supplementary table S3, Supplementary Material online). These included *atpA*, *atpB*, *petB*, *petD*, *psaA*, *psaB*, *psbA*, *psbB*, *psbC*, *psbD*, *psbE*, *psbI*, *psbT*, plastid 23S ribosomal DNA(*cp23S rDNA*), plastid 16S ribosomal DNA (*cp16S rDNA*). Furthermore, four noncoding minicircles (contig 14, 20, 21, 27) were obtained by combining Illumina and PCR-based data. In addition, we obtained other two contigs (contig 18, 26) that could not form circle based on our dataset.

### Minicircles Copy Number in *F. kawagutii*

To compare copy number differences among different minicircles, the sample collected at 12 h (2 hours before the end of the light period) in the exponential growth stage was selected as template, and digital PCR was performed to quantify the copy number of each minicircle per cell. Only the 15 minicircles with functionally annotatable genes were analyzed (Table 1). Results showed that the copy number of these 15 minicircles varied between 3.89 and 34.3 copies/cell. The gene with the highest copy number was *psbT*, and the gene with the lowest copy number was *psbI*.

**Table 1:** The copy numbers of *Fugacium kawagutii* minicircles estimated at 12 h using ddPCR

Gene name	*atpA*	*atpB*	*psaA*	*psaB*	*psbA*	*psbB*	*psbC*	*psbD*	*psbE*	*psbI*	*psbT*	*petB*	*petD*	*cp23S rDNA*	*cp16S rDNA*
Copies/cell	4.63 ± 0.13	13.29 ± 0.4	4.95 ± 0.54	13.35 ± 0.61	11.46 ± 0.15	13.76 ± 1.82	23.35 ± 0.67	10.56 ± 0.6	21.95 ± 0.78	4.46 ± 0.57	28.76 ± 5.54	9.59 ± 0.3	8.32 ± 1.34	16.83 ± 0.43	12.22 ± 1.96

### The Minicircles Replication Dynamics in the Light/Dark Cycle

**Fig. 2.**
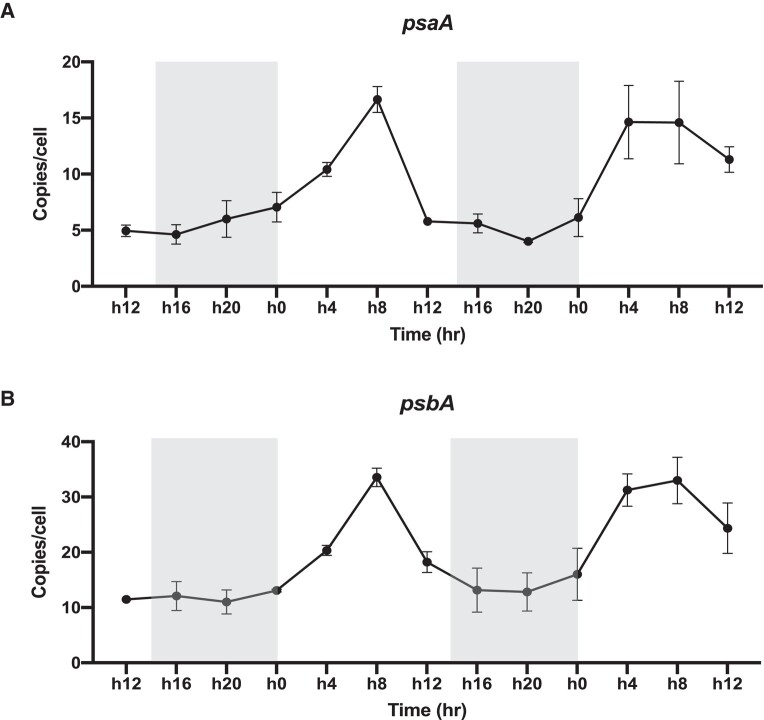
The diel dynamics of copy numbers of *psaA* (a) and *psbA* (b) over a 48 h sampling period in the 14:10 light:dark cycle. Grey shading indicates dark period.

A gene from photosystem I (*psaA*) and another from photosystem II (*psbA*) were used in the analysis of replication dynamics in the light/dark cycle using digital PCR. The results showed that copy number of *psaA* and *psbA* gene varied through the light/dark cycle, in a consistent trend across two light/dark cycles that we examined. In the two light/dark cycles, the copy number of these two genes changed on average from 3.6 to 18.8 copies/cell and from 9 to 37.5 copies/cell, respectively (Fig. 2). Under both light/dark cycles, the gene copy number showed an upward trend after 4 h in the light, reached its highest value after 8 h in the light and finally declined after 12 h in the light. Overall, *psaA* and *psbA* replicated 2 to 5 times on average in each light period.

### GC Contents and Sequence Lengths of Minicircles

We found that the GC content of the whole *F. kawagutii* plastid genome was 40%, which is between that in *H. triquetra* (38%) and that in *Clade C3* (42%) (Table 2). Comparatively speaking, the length of individual Symbiodiniaceae (*Clade C3* and *F. kawagutii*) minicircles are smaller than that in non Symbiodiniaceae dinoflagellates (*T. horridus*, *H. triquetra, A. carterae* and *A. operculatum*) except the *atpB, psaA,* and *psaB* minicircles (Table 2). Furthermore, for most minicircles (*atpA*, *petB*, *psaB*, *psbA*, *psbC*, *psbD*, *psbE*, *psbI*, *psbT*, *cp16S rDNA*, *cp23S rDNA*), those in *F. kawagutii* are the shortest. Besides, the *atpA, petB, psaA, psaB, psbC, psbD* and *psbE* minicircles have the shortest CDS length in *F. kawagutii* compared to other five species.

**Table 2:** Characteristics of plastid minicircles in *Fugacium kawagutii* and the other dinoflagellate chloroplast genomes studied to date^a^

	Tripos horridus	Heterocapsa triquetra	Amphidinium carterae	Amphidinium operculatum	Symbiodinium sp. Clade C3	Fugacium kawagutii
Gene minicircles						
*atpA*	…	2,444/1,359	2,606/1,395	2,713/1,377	2,213/1,467	2,095/1,266
*atpB*	…	…	2,587/1,647	2,483/1,647	2,691/1,710	2,603/1,878
*petB*	…	2,204/660	2,606/660	2,713/660	1,722/657	1,601/408
*petD*	…	2,188/477	2,567/474	2,416/474	1,419/477	1,436/477
*psaA*	…	3,005/2,199	…	2,443/2,016	2,788/2,022	2,752/1,794
*psaB*	6,266/2,203	3,121/2,331	…	2,363/1,965	2,741/2,082	2,326/1,716
*psbA*	…	2,151/1,047	2,556/2,037	2,311/1,023	1,914/1,029	1,833/1,257
*psbB*	5,853/1,457	2,286/1,518	2,426/1,521	2,282/1,521	2,232/1,494	2,246/1,533
*psbC*	5,917/1,418	2,330/1,383	2,477/1,380	2,341/1,380	2,135/1,359	1,744/975
*psbD*	6,696/1,046	2,628/1,068	2,369/1,068	2,358/1,068	1,880/1,074	1,842/807
*psbE*	1,959/229	2,196/234	2,369/234	2,358/234	1,309/234	1,227/231
*psbI*	…	…	2,369/108	2,358/108	…	1,580/108
*psbT*	…	…	…	…	…	1,101/84
LSU-rRNA	…	3,027	…	…	2,839	2,802
SSU-rRNA	…	2,563	2,651	2,651	1,420^b^	1,696
Noncoding No./total length	6/35,702	1plus 5 chimeric/12,684	10/18,196	5/7,367	0	4/4,674
Total G + C content (%)	35%	38%	45%	46%	42%	40%
Core region	…	106	70	49	84	94
Total length	62,393	42,827	38,435	31,728	27,303	33,558

^a^Values depict the length (bp) of the minicircle/gene or core region. Noncoding No./total length indicates the number of gene-free minicircles/sum of those minicircles length.

^b^Partial cp16S rRNA,/in gene minicircles (total length/CDS length).

### Coding Region of Minicircles

In the coding region of minicircles in *F. kawagutii*, the most abundant three amino acids in the minicircle-encoded proteins are leucine (Leu), serine (Ser) and alanine (Ala), as is also true in *A. carterae*, *A. operculatum* and *Clade C3*. Furthermore, we found that of all the investigated species, the top three amino acids accounted for approximately 30% of all amino acids (supplementary table S4, Supplementary Material online). In addition, *F. kawagutii* uses TGA, TAG and TAA as stop codons, the same as *T. horridus* and *A. carterae* (supplementary table S4, Supplementary Material online). This contrasted the other symbiodiniacean species *Clade C3*, which only uses TGA and TAA as stop codons. We then counted rare codons (occurring 10 or fewer times) according to (Barbrook et al. 2014). In the Symbiodiniaceae (*F. kawagutii* and *Clade C3*), only seven rare codons were observed, compared to other four dinoflagellate species examined (supplementary fig. S1, Supplementary Material online).

### Noncoding Region of Minicircle

**Fig. 3.**
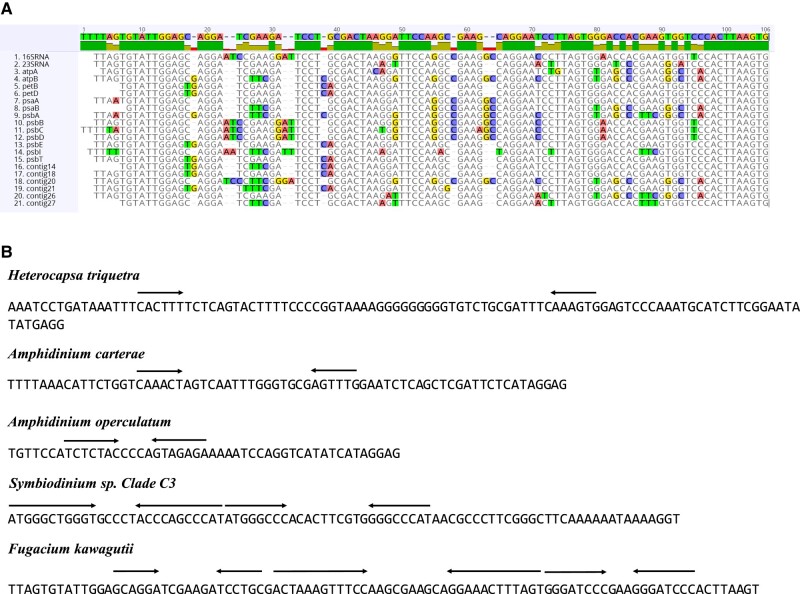
The core regions in five dinoflagellates. a) Alignment of the core regions in *Fugacium kawagutii* plastid minicircles. b) The inverted repeats of plastid core regions in five species of dinoflagellates. Arrows indicate inverted repeats.

In order to identify conserved sequences in the noncoding region of all minicircles, the sequences of *F. kawagutii* minicircles were aligned and compared (Fig. 3A). The alignment showed that all minicircle contigs had a highly conserved core sequence, 94 bp in length, which only had occasional single base differences. We propose that this is the core region of *F. kawagutii* plastid minicircles. There are three pairs of short inverted repeat sequences in the *F. kawagutii* core region (Fig. 3B).

### Codon Usage Bias of Peridinin Dinoflagellate Plastid Genomes

**Fig. 4.**
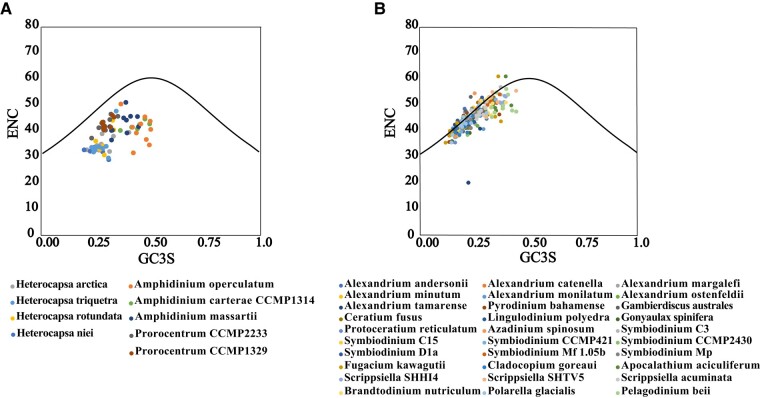
ENc-plot analysis (ENc plotted against GC_3S_). ENc denotes the effective number of codons, and GC_3S_ denotes the GC content on the third synonymous codon position. The expected ENc from GC_3S_ are shown as a standard curve. a) The ENc-plot analysis of Heterocapsaceae, Amphidiniaceae and Prorocentraceae. b) The ENc-plot analysis of Pyrocystaceae, Ceratiaceae, Lingulodiniaceae, Gonyaulacaceae, Protoceratiaceae, Amphidomataceae, Symbiodiniaceae, Thoracosphaeraceae, Peridiniaceae, Suessiaceae.

The Effective Number of Codons (ENc) against GC_3S_ (ENc-GC_3S_) plot was generated for each coding sequence to investigate the role of mutational pressure in shaping codon usage bias. When codon bias is affected only by mutation, the observed ENc values should be distributed along or close to the standard curve, but when codon bias is affected by selection and other factors, the observed ENc values will deviate from the standard curve (Wang et al. 2018). As shown in (Fig. 4, supplementary fig. S2, Supplementary Material online), the results of ENc-plot analysis for dinoflagellate species in the same family were similar. More specifically, Heterocapsaceae, Amphidiniaceae, and Prorocentraceae species plastid genes, their observed ENc values are far off from the standard curve (Fig. 4A, supplementary fig. S2(A-C), Supplementary Material online), indicating that the codon usage bias in the three families are mainly affected by selection factors. However, some plastid genes of the observed ENc values of other dinoflagellate lineages (Pyrocystaceae, Ceratiaceae, Lingulodiniaceae, Gonyaulacaceae, Protoceratiaceae, Amphidomataceae, Symbiodiniaceae, Thoracosphaeraceae, Peridiniaceae, Suessiaceae) are located on or near the standard curve (Fig. 4B, supplementary fig. S2(D–M), Supplementary Material online), indicating that the codon usage bias of these genes are affected by mutation pressure.

**Fig. 5.**
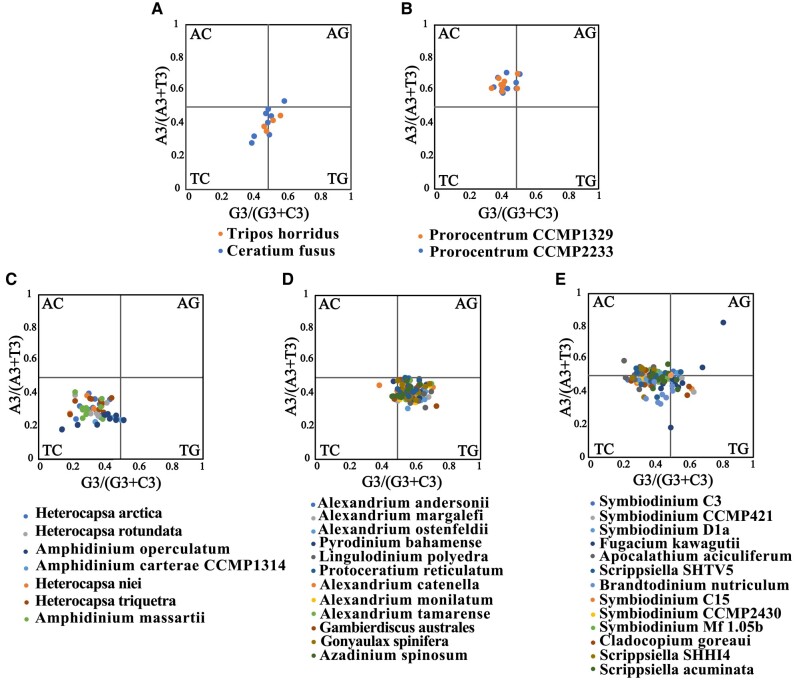
Parity rule 2 (PR2)-bias plot. [A_3_/(A_3_ + T_3_) against G_3_/(G_3_ + C_3_)]. a) The PR2-plot analysis of Ceratiaceae. b) The PR2-plot analysis of Prorocentraceae. c) The PR2-plot analysis of Heterocapsaceae, Amphidiniaceae. d) The PR2-plot analysis of Pyrocystaceae, Lingulodiniaceae, Gonyaulacaceae, Protoceratiaceae, Amphidomataceae. e) The PR2-plot analysis of Symbiodiniaceae, Peridiniaceae, Thoracosphaeraceae.

The Parity rule 2 (PR2) analysis was also used to estimate the effects of natural selection and mutation pressure on codon usage bias according to the preferential base in the third position (Fig. 5, supplementary fig. S3, Supplementary Material online). Generally, if only mutation pressure is at play, A_3_ = T_3_ and G_3_ = C_3_; if both natural selection and mutation pressure are at play, A_3_ ≠ T_3_ and G_3_ ≠ C_3_ (Sueoka 1995, 1999). The base preference is consistent across species in the same family. Specifically, the PR2 bias plot in Heterocapsaceae and Amphidiniaceae prefer T and C (Fig. 5C, supplementary fig. S3(A-B), Supplementary Material online); Prorocentraceae prefer A and C (Fig. 5B, supplementary fig. S3C, Supplementary Material online); Pyrocystaceae, Lingulodiniaceae, Gonyaulacaceae, Protoceratiaceae and Amphidomataceae prefer T and G (Fig. 5D, supplementary fig. S3(D, F-I), Supplementary Material online); Symbiodiniaceae, Peridiniaceae and Thoracosphaeraceae prefer C (Fig. 5E, supplementary fig. S3(J-L), Supplementary Material online); Ceratiaceae prefer T (Fig. 5A, supplementary fig. S3E, Supplementary Material online). The PR2-plot analysis results indicated that these dinoflagellates codon usage is not balanced and affected by both natural selection and mutation pressure.

Combining the results of the two analyses, the factors influencing codon usage bias in dinoflagellates can be divided into two categories. The first category is represented by Heterocapsaceae, Amphidiniaceae and Prorocentraceae with codon usage bias being mainly affected by natural selection pressure. The second category is represented by Pyrocystaceae, Symbiodiniaceae, Peridiniaceae and Ceratiaceae, with codon usage bias being affected by both natural selection pressure and mutation pressure.

### Phylogenetic Tree of Plastid-encoded Proteins

**Fig. 6.**
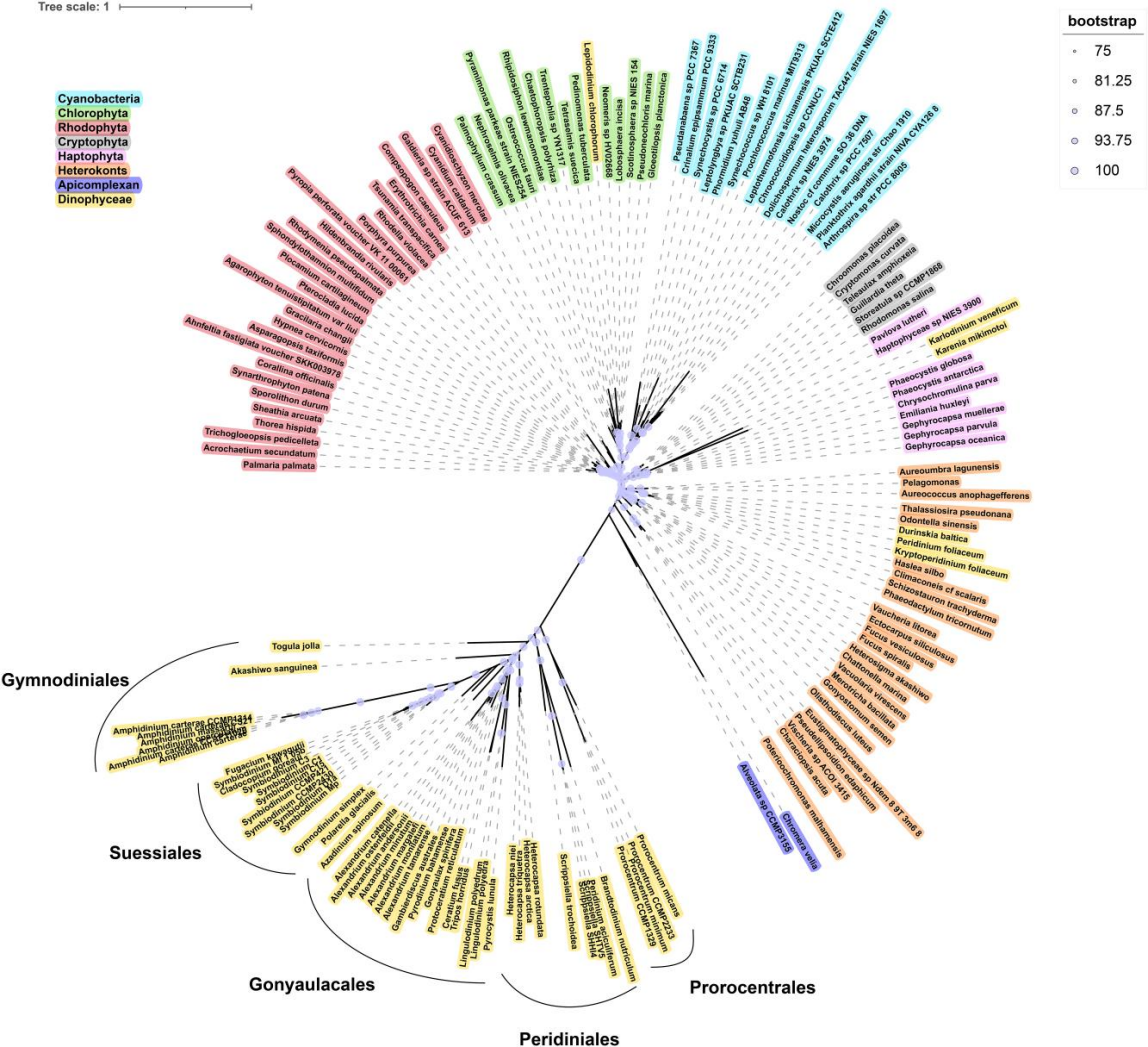
Multi-protein phylogeny of plastid-encoded proteins. Tree topology shown is from maximum-likelihood (ML) analysis based on 12 plastid-encoded protein sequences. Circles at each node indicate support of nodes that is >75% in ML bootstrap values. Taxonomic groupings of dinoflagellates and other photosynthetic protists, apicomplexan and cyanobacteria are shown in different colors. Dinoflagellate orders (Gymnodiniales, Gonyaulacales, Peridiniales, Suessiales, and Prorocentrales) are labeled on the diagram.

A plastid-encoded protein phylogenetic tree (Fig. 6) was constructed using a concatenated protein alignment, consisting of the 12 plastid-encoded proteins (ATPA, ATPB, PETB, PETD, PSAA, PSAB, PSBA, PSBB, PSBC, PSBD, PSBE, PSBI) sequences studied so far covering 154 representative species (supplementary table S1, Supplementary Material online) from eight phyla/classes. All typical (peridinin-containing) species of dinoflagellates were clustered together. However, the nontypical species, including *Lepidodinium chlorophorum*, *Karlodinium veneficum*, *Karenia mikimotoi*, *Durinskia baltica*, *Kryptoperidinium foliaceum* and *Peridinium foliaceum* formed separate clusters because their plastids were secondary or tertiary replacements plastids. The plastid of *L. chlorophorum* was derived from green algae (Kamikawa et al. 2015), whereas *K. veneficum* and *K. mikimotoi* species have plastids from haptophyte (Tengs et al. 2000). The plastids of *K. foliaceum*, *D. baltica* (Imanian et al. 2010) and *P. foliaceum* (Yoon et al. 2005) were derived from diatoms. These exceptions were species in the order of Gymnodiniales (*L. chlorophorum*, *K. veneficum*, *K. mikimotoi*) and Peridiniales (*D. baltica*, *K. foliaceum*, *P. foliaceum*).

In the tree, the Amphidiniaceae species have the longest branches in the Dinophyceae lineage, and the branches in the Dinophyceae lineage were longer than that in other phyla/classes, but surprisingly, although the plastid-encoded proteins of *K. veneficum* and *K. mikimotoi* clustered with haptophytes, the length of the evolutionary branch were much longer than haptophytes species. This probably indicated that plastids in the dinoflagellate intracellular environment (at least for these 52 species) evolved faster than in other algal intracellular environments.

### Phylogenetic Tree of Nucleus-encoded Plastid Proteins

Five nucleus-encoded plastid proteins (ATPI, ATPH, PSAD, PSBF, PSBV) sequences were concatenated for 112 species (supplementary table S1, Supplementary Material online). These genes were identified by protein sequences containing plastid signal peptides in the peridinin dinoflagellate, and there is no information about these five genes in peridinin dinoflagellate plastid genomes. But in other phyla/classes, they remain in the plastid. These 112 species corresponded to species in the plastid-encoded protein phylogenetic tree.

Similar to the plastid-encoded protein tree, in the nucleus-encoded plastid protein tree (supplementary fig. S4, Supplementary Material online), species from the same phyla/classes tended to cluster together, except for the six species with replacement plastids (*L. chlorophorum*, *K. veneficum*, *K. brevis*, *K. mikimotoi*, *G. baltica*, *K. foliaceum*), which were also branched similarly to the branching pattern in the plastid-encoded protein tree. The length of the evolutionary branch was much longer in *K. veneficum, K. mikimotoi* and *K. brevis* in comparison to haptophyte species. In addition, the result also showed that after the plastid genes transferred into the nucleus in Dinophyceae, these five plastid-targeted proteins in peridinin dinoflagellates (at least for these seven species) evolved much faster than their counterparts in other eukaryotic algae (particularly red alga descendants), in which these genes are plastid-encoded.

## Discussion

Given the importance of dinoflagellates in the coral reef and other ecosystems in the ocean and the unique characteristics of their plastid genomes, the effort to study plastid genomes in dinoflagellates has been inadequate. Only seven species of dinoflagellates plastid genome have been reported previously. These studies have revealed the gene content, structural features, 3′ polyU tail, 5′ end processing and RNA editing of the minicircles (Barbrook et al. 2019). Here we report the plastid genome of the eighth species of dinoflagellates, documenting its copy number and its replication rhythm. In addition, our comparative analysis also unveils shared and unique characteristics of the plastid genome in dinoflagellates. Finally, we report the inferred evolutionary trend of plastid proteins in dinoflagellates relative to other algae.

### The Features, Copy Number and Replication Rhythm of *F. Kawagutii* Minicircles

Through comparative analysis, we found that the minicircles of *F. kawagutii* maintains the characteristics of typical minicircles. However, compared to other five dinoflagellate species, *F. kawagutii* minicircles are either the shortes (*atpA*, *petB*, *psaB*, *psbA*, *psbC*, *psbD*, *psbE*, *psbI*, *psbT*, *cp16S rDNA*) or have the shortest coding sequences (*atpA, petB, psaA, psaB, psbC, psbD,* and *psbE*) (Table 2). A recent genomic analysis of the same strain predicted plastid genes and the core region (Liu et al. 2018). However, the core region in that study is not the same with the core region identified in the present study, which is consistent across all minicircles in this species. This discrepancy suggests that isolating and sequencing plastid genomes (with no or minimal nuclear DNA interference) may be preferred in identifying plastid genes and core regions.

Besides, in *F. kawagutii*, we identified *psbT* in minicircles, the first documentation of this gene in Symbiodiniaceae. The *psbT*-encoded protein seems to play a role in the dimerization of PSII (Fagerlund et al. 2020). The important function suggests that this gene might exist in all species of Symbiodiniaceae but has escaped detection in the previously studied species. This was detected probably because we isolated plastids for sequencing whereas previous studies extracted total DNA, which was then subjected to CsCl density gradient centrifugation to separate circular DNA (Zhang et al. 1999; Barbrook and Howe 2000; Barbrook et al. 2001; Hiller 2001; Koumandou et al. 2004; Zauner et al. 2004; Barbrook et al. 2006; Barbrook et al. 2014; Liu 2015) or PCR amplification of the minicircles for sequencing (Adrian C. Barbrook et al. 2014; Xiaojuan Liu 2015). It is also possible that the copy number of *psbT* is higher in *F. kawagutii* making it easier to isolate.

The copy number of *F. kawagutii* minicircles largely fell in the 3.89 to 34.3 copies/cell range in exponential growth phase (Table 1). Previous research has shown a wider range of copy numbers per cell in different growth stages in dinoflagellates. In *A. operculatum*, the copy numbers of the five minicircles previously examined (*atpB*, *petD*, *psbB*, *psbD/E/I*, *cp23S rDNA*) were found to be low (2 to 4 copies/cell) during the exponential growth stage but to increase (50 to 420 copies/cell) during the later growth phase (Koumandou and Howe 2007). These data combined indicate a strong dynamics of minicircle copy number throughout the culture growth cycle.

There have been no reports on how the replication of minicircles occurs in a diel cycle, but our results reveal a strong diel dynamics. *F. kawagutii* minicircles began to replicate upon the onset of the light period (Fig. 2). This pattern seems to be common in cpDNA. In plants, light is an important factor during plastid development to regulate cpDNA replication, structure and stability (Shaver et al. 2008; Zheng et al. 2011; Chen et al. 2021a). In the green alga *Chlamydomonas reinhardtii*, it was also found that the replication of cpDNA was positively correlated with the light period duration (Kabeya and Miyagishima 2013). Our experimental results also show that the copy number of minicircles per cell reached the peak after light was turned on for eight hours, and then gradually returned to the average level in the dark period. According to previous reports, the decline is probably due to cell division and plastid DNA degradation that occurs to maintain proper DNA load per plastid (i.e. per cell) (Matsushima et al. 2011; Kabeya and Miyagishima 2013; Sakamoto and Takami 2018).

### General Features of Peridinin-type Dinoflagellate Plastid Genome

Among the numerous phylogenetically diverse dinoflagellate lineages, plastid genomes have been examined for species from four taxonomic orders (Gymnodiniales, Suessiales, Gonyaulacales, Peridiniales), and in all cases were found in minicircles. Whether any of the replacement plastids (serial secondary endosymbiosis and tertiary replacement) is minicircularized is unclear, it appears that the peridinin-type plastid genomes all exist as minicircles. Major characteristics of the peridinin-type dinoflagellate plastid genomes have been previously documented (Howe et al. 2008; Barbrook et al. 2014). With the addition of the *F. kawagutii* plastid genome and a comparative analysis, some previously unnoticed features emerged.

First, GC content and minicircle lengths are similar in peridinin dinoflagellate plastid genomes. Based on all the plastid genomes that have been sequenced to date, the total GC content are in the range of 35% to 46% (Table 2), which are comparable to the GC content of nondinoflagellate algal plastid genomes (Ren et al. 2021). Except for *T. horridus*, the lengths of other individual minicircles are mostly around 1,000 to 3,000 bp (Table 2).

Second, several codons are most frequently used in the coding region of minicircles. In the six peridinin dinoflagellate plastids that have been investigated, the most frequent amino acids encoded in minicircles are Leu, Ala, Ser, and Gly, and the top three amino acids account for about 30% of the total amino acids (supplementary table S4, Supplementary Material online). Interestingly, this amino acid distribution pattern is similar to that in both plant plastid genomes and animal mitochondrial genomes (Chen et al. 2021b; Trofimov et al. 2022). Furthermore, the codon preferences of genes with T-ending codons are conserved among minicircles (supplementary fig. S3, Supplementary Material online). Several studies have indicated that high AT content is the main reason for synonymous codons ending in A/T (Shimada and Sugiura 1991; Clegg et al. 1994), which might have resulted from natural selection and mutation during evolution (Liu et al. 2019). The types of rare codons are not highly overlapping among the six species (supplementary fig. S1, Supplementary Material online). Rare codons can influence the gene translation, especially lowering the rate of translation, causing errors and impacting the speed of protein synthesis and stability (Gingold and Pilpel 2011; Liu et al. 2021). This result is consistent with a previous report on three dinoflagellate species (Barbrook et al. 2014).

Third, the core region of the minicircles is highly conserved in Symbiodiniaceae (at least for *Cladocopium* and *Durusdinium*) but can be highly variable in A*. carterae*. Previous studies have shown that the noncoding region of minicircles contains a highly conserved core region, and the core region is variable in length between species (Zhang et al. 1999; Barbrook et al. 2001; Zhang et al. 2002; Koumandou et al. 2004). All core regions commonly contain inverted repeats (Fig. 3B), and the inverted repeats are related to initiation of replication and termination of transcription (Stauffer and Stauffer 1988; Chew et al. 2005; Leung et al. 2005). Thus, it has been speculated that the core region are responsible for the maintenance of the copy number, the initiation of replication and the transcription of minicircles (Zhang et al. 1999; Barbrook and Howe 2000; Barbrook et al. 2001; Hiller 2001; Zhang et al. 2002). In the present study, the core regions are conserved within and between species within a symbiodiniacean genus (supplementary fig. S5, Supplementary Material online). In contrast, it is striking that the core regions within the non Symbiodiniaceae dinoflagellate A*. carterae* are highly diverse across different minicircles (Liu 2015). Strikingly, the degree of divergence between plastid minicircle core regions within *A. carterae* is comparable to that between minicircle core regions from different genera in Symbiodiniaceae.

Fourth, there seems to be variability in the features, codon usage bias and pace of evolution of minicircles in different lineages of dinoflagellates. In the minicircles of Amphidiniaceae, the core regions are in general highly divergent (Liu 2015), the gene load on minicircles ranges one to three genes (although one is most common) per minicircle (Howe et al. 2008), and the total GC content is particularly high (probably because the codon ends prefer T/C). Our ENc-plot and PR2-plot analyses show the Amphidiniaceae codon bias are mainly affected by natural selection pressure. Both the results of the core regions (noncoding region) analysis and the coding region phylogenetic tree show that Amphidiniaceae have higher rates of plastid evolution than other peridinin dinoflagellates. In Symbiodiniaceae, the core regions of the same genus are much more conserved, each minicircle only contains one gene and the length of individual minicircles in *Clade C3* and *F. kawagutii* are smaller compared to other four dinoflagellates (*T. horridus*, *H. triquetra, A. carterae,* and *A. operculatum*) except the *atpB, psaA,* and *psaB* minicircles. Furthermore, our ENc-plot and PR2-plot analyses show that codon bias in Symbiodiniaceae are affected by both natural selection pressure and mutation pressure, and the ending of codons in minicircles prefers C. The minicircles of the family Ceratiaceae seems on the other end of the spectrum: they are the largest among all identified dinoflagellate minicircles, but their total GC contents are the lowest with their ending of codons preferring T, and our ENc-plot and PR2-plot analyses show that the codon bias are affected by both natural selection pressure and mutation pressure. In the family Heterocapsaceae, the ending of codons in minicircles prefer T and C, each minicircle only contains one gene and our ENc-plot and PR2-plot analyses show that codon bias are mainly affected by natural selection pressure.

### Accelerated Evolution of Dinoflagellate Plastid Proteins Were Probably Driven by the Dinoflagellate Environment

Previous studies have found evidence that the plastid-encoded protein of the peridinin dinoflagellates have accelerated evolution (Zhang et al. 2000; Bachvaroff et al. 2006; Barbrook et al. 2014). In the present study, our analyses confirm that, but further indicate that the accelerated evolution has also occurred in nucleus-encoded plastid proteins. Furthermore, as shown in our phylogenetic trees, not only the plastid proteins of peridinin dinoflagellates evolved faster than that in other algal lineages, but the plastid proteins of haptophyte-origin (fucoxanthin replacing peridinin) tertiary replacement plastids (at least for *K. veneficum, K. mikimotoi* and *K. brevis*) as well show accelerated evolution relative to the plastid proteins in haptophyte algae (Fig. 6, supplementary fig. S4, Supplementary Material online). Evidently, the dinoflagellate intracellular environment probably driven a faster evolution of the acquired plastid genomes, regardless of origin of the plastids and current location (nucleus vs. plastid) of the coding genes.

The faster pace of evolution makes the plastid protein genes potentially useful for resolving closely related lineages of dinoflagellates. The 18S ribosomal subunit (18S rRNA) gene has been the most widely used genetic marker for phylogeny because of the large number of sequences available in public repositories (Heldal et al. 2013; John et al. 2014; Mordret et al. 2018). But using 18S rRNA cannot correctly resolve all species of the same order in dinoflagellates. For example, in the 18S rRNA phylogenetic tree, the genera *Ceratium*, *Tripos*, *Lingulodinum*, *Gonyaulax*, *Protoceratium* and *Alexandrium* in Gonyaulacales do not form a monophyletic group. Similarly, the *Heterocapsa*, *Scrippsiella*, and *Peridinium* are not clustered together in the Peridiniales order (Mordret et al. 2018). In our analysis, the above-mentioned lineages form monophyletic clades in the phylogenetic tree of plastid-encoded proteins (Fig. 6). Plastid protein genes perform better in distinguishing different orders in dinoflagellates. Besides, past research has found that the noncoding region of *psbA* is the fastest evolving genetic marker known for Symbiodiniaceae, and can be a useful gene marker for ecology, evolutionary biology, and systematics of symbiotic dinoflagellates (LaJeunesse and Thornhill 2011). Therefore, the potential of minicircles (including coding and noncoding regions) as DNA barcodes and for phylogenetic studies of dinoflagellates, particularly the hard-to-resolve coral-symbiont family Symbiodiniaceae (Davies et al. 2023), should be explored further in future research.

## Materials and Methods

### Alga Cultures

The culture of *F. kawagutii* (strain CCMP2468) was from the National Center of Marine Algae and Microbiota (NCMA), Bigelow Laboratory for Ocean Sciences (East Boothbay, ME, USA). The culture was grown in L1-medium (Guillard and Hargraves 1993) prepared using surface seawater collected from northern South China Sea, which was 0.22 μm filtered and autoclaved. Temperature was controlled at 26 °C and illumination was provided at 200 μE·m^−2^ s^−1^ under a 14 h:10 h light:dark cycle. The onset of the light period was designated as h 0.

### Plastid Isolation and Genome Extraction

The algal cells in the late logarithmic phase were collected by centrifugation (5,000 rpm, 15 min) and broken using the Fastprep®-24 Sample Preparation System (MP Biomedicals, USA) with bead-beating (3:1 mixture of 0.5 and 0.1 mm diameter ceramic beads) as previously reported (Yuan et al. 2015). The sample was resuspended in 1 ml Buffer A (50 mM HEPES, 300 mM Sorbitol, 5 mM MgCl_2_, 50 mM EDTA, 5 mM C_6_H_7_O_6_Na, 1% β-Mercaptoethanol, pH 7.5 to 7.8 at 4 °C) and the unbroken cells were removed by centrifugation at 1,800 rpm for 10 min at 4 °C. The plastid was collected by centrifugation at 5,000 rpm for 10 min at 4 °C. The plastid pellet was resuspended in the Buffer B (50 mM HEPES, 300 mM Sorbitol, 5 mM MgCl_2_, 50 mM EDTA, 5 mM C_6_H_7_O_6_Na, pH 7.5 to 7.8 at 4 °C) and loaded onto 50% Percoll density gradient in the Buffer B by centrifugation at 3,000 × *g* for 20 min at 4 °C to collect the sample above 50%. The collected samples were loaded onto 15% Percoll density gradient in the Buffer B by centrifugation at 3,000 × g for 20 min at 4 °C to collect the sample below 15%. The enriched plastid was confirmed by observing auto-fluorescence at 420 nm. Next the cpDNA extraction was then conducted using a CTAB protocol coupled with Zymo DNA Clean & concentration kit (Zymo Research Corp, Irvine, USA) following (Yuan et al. 2015). The quality of genomic DNA was checked on 1% agarose gel for a single intact band. 1μl of the sample was used for determining the DNA concentration by NanoDrop™ 2000 Spectrophotometer (Thermo Scientific™). The purified cpDNA was supplied to Genewiz (Genewiz, China) for sequencing the plastid genome of *F. kawagutii*.

### Plastid Genome Sequencing, Assembly and Annotation

Next generation sequencing library preparations were constructed following the manufacturer's protocol. About 200 μg genomic DNA was randomly fragmented by Covaris to an average size of 300 to 350 bp. The fragments were treated with End Prep Enzyme Mix for end repairing, 5′ phosphorylation and 3′ adenylated, to add adaptors to both ends. Size selection of Adaptor-ligated DNA was then performed by DNA Cleanup beads. Each sample was then amplified by PCR for eight cycles using P5 and P7 primers, with both primers carrying sequences which can anneal with the flowcell to perform bridge PCR and P7 primer carrying a six-base index allowing for multiplexing. The PCR products were cleaned up and validated using an Agilent 2100 Bioanalyzer. The qualified libraries were sequenced pair end PE150 on the illumina Novaseq System.

The sequencing reads were quality-filtered to remove adaptors and low-quality reads. The resulting high-quality reads were then assembled using velvet or Novoplasty, gapfilled with SSPACE and GapFiller (Zerbino and Birney 2008; Zerbino et al. 2009; Boetzer et al. 2011; Boetzer and Pirovano 2012; Hunt et al. 2014). Using the assembled sequences, new “outward” primers were designed to amplify the remainder of the minicircle sequences. The “outward” PCR amplification of minicircles followed a previous report (Barbrook et al. 2014)

Coding genes were identified using Prodigal (for plasmid) (Delcher et al. 2007), MITOS2 (for Animal mitochondria) (Stanke et al. 2006) and GoSeq (for Plant mitochondria) (Tillich et al. 2017) gene-finding software. Transfer RNAs (tRNAs) were detected in the genome using the program tRNAscan-SE (Lowe and Eddy 1997) with default parameter settings. rRNA genes were identified by using RNAmmer (Lagesen et al. 2007). The coding genes were annotated with National Center for Biotechnology Information (NCBI) nr database by BLAST. Then the functions of genes were further annotated by GO (Gene Ontology Consortium 2004) (Gene Ontology) database, and the pathways were annotated using KEGG (Kanehisa and Goto 2000) (Kyoto Encyclopedia of Genes and Genomes) database.

The *Cladocopium*, *Durusdinium* and *Amphidinium carterae* noncoding minicircle sequences in supplementary fig. S5, Supplementary Material online were collected from GenBank (http://www.ncbi.nlm.nih.gov) and Liu (Liu et al. 2018).

### Phylogenetic Analyses

To reconstruct the phylogenetic relationship among dinoflagellates, a set of 159 plastid protein sequences were collected from GenBank (http://www.ncbi.nlm.nih.gov), Sampgr (http://sampgr.org.cn) and the Marine Microeukaryote Transcriptome Sequencing Project (MMETSP) (Keeling et al. 2014) sorted by Dorrell (Dorrell et al. 2017) (listed in supplementary table S1, Supplementary Material online). The alignment for the concatenated sequences was done using MAFFT (Katoh and Toh 2008). Subsequently, the dataset was analyzed using the maximum-likelihood (ML) on the W-IQ-Tree web server (Trifinopoulos et al. 2016). Plastid signal peptides were predicted using TargetP-2.0 web servers (https://services.healthtech.dtu.dk/services/TargetP-2.0/) (Nielsen et al. 1997; Emanuelsson et al. 2000).

### Effective Number of Codons (ENc) and ENc-plot Analysis

Forty plastid gene sequences were collected from GenBank (http://www.ncbi.nlm.nih.gov), Sampgr (http://sampgr.org.cn) and the Marine Microeukaryote Transcriptome Sequencing Project (MMETSP) (Keeling et al. 2014; Caron et al. 2017) (listed in supplementary table S2, Supplementary Material online).The effective number of codons (ENc), the indicator of the degree of codon usage bias and the extent of preference of synonymous codons (Liu et al. 2010), was calculated by the “chips” program in European molecular biology open software suite (EMBOSS) explorer (http://www.bioinformatics.nl/emboss-explorer/), The GC_3s_ contents were obtained using the EMBOSS explorer “cusp” program.

We carried out ENc-plot analysis, which is the plotting of ENc values versus GC_3S_. ENc-plot analysis is normally used to discover factors inducing the codon usage patterns (Wu et al. 2020). The expected ENc values of GC_3S_ were calculated as (ENc = 2 + GC_3S_ + 29/(GC_3S_^2^ + (1 – GC_3S_)^2^) (Fuglsang 2006). ENc-GC_3S_ plots were generated using R (version 3.6.2) following R Core Team (2021).

### Parity Rule 2 (PR2) Plot Analysis

Using the same 40 plastid gene sequences as those in the ENc-plot analysis described above, PR2 analysis was conducted to estimate the effects of natural selection and mutation pressure on codon usage. The ordinate was [A_3_/(A_3_ + T_3_)] value, and the abscissa was [G_3_/(G_3_ + C_3_)]. The PR2-plot was drawn using Excel.

### Sample Collection for Light/Dark Cycle and DNA Extraction

When the *F. kawagutii* cultures were in the exponential growth phase, we conducted a diel sampling campaign for a 48-h period. Three independent cultures were sampled every 4 h, starting two hours before the onset of the dark period. At each time point, 2 × 10^5^ cells were collected as described above for DNA extraction.

For DNA extraction, 5% Chelex buffer (Chelex 100, Molecular Biology Grade Resin, Bio-Rad, Hercules, CA, USA) was added to the cell pellet. The suspension was mixed on a vortex machine and the mixture was incubated for 20 min at 97 °C (Nagai et al. 2012; Yarimizu et al. 2021). Then cells were homogenized using the Fastprep®-24 Sample Preparation System with bead-beating, run for three cycles each at the rate of 6 m s^−1^ for 1 min to ensure that the cells are completely disrupted. The cell lysate was then centrifuged at 3,000 rpm for 10 min, and the supernatant was collected into a clean Eppendorf tube for digital PCR.

### Droplet Digital PCR

Detection and quantification of minicircles in the extracted DNA was performed using the droplet digital PCR (ddPCR) platform. The ddPCRs were performed with the QIAcuity 5-plex System (Qiagen, Germany) in 8.5 K (24-well) Nanoplates (Qiagen, Germany). The ddPCR was carried out in a 12 μl reaction mixture contained: 4 μl 3× EvaGreen PCR Master Mix, 0.48 μl each of the forward and reverse primers (10 μM), 4.04 μl nuclease-free water, and 3 μl template DNA. Every primer described here is detailed in supplementary table S5, Supplementary Material online. The thermal cycling conditions were as follows: initial heat activation (95 °C for 2 min), 40 cycles of denaturation (95 °C for 15 s), annealing (55 °C for 15 s), and extension (72 °C for 15 s), and cooling (40 °C for 5 min). Absolute quantification and CNV were estimated with QIAcuity Software Suite 2.0.20 (Qiagen). EvaGreen detection was done using the green channel with 500-ms exposure and gain 6. After endpoint PCR, the starting concentration of template is determined by Poisson statistical analysis of the number of positive (containing amplified target) and negative (no amplified target detected reactions) (Hindson et al. 2013).

## Supplementary Material

Additional supplemental materials may be found online in the Supporting Information section. Supplementary material is available at *Genome Biology and Evolution* online.

## Supplementary Material

evad237_Supplementary_Data

## Data Availability

Raw sequence data are deposited in the Zenodo. https://doi.org/10.5281/zenodo.10275789.
