# Supplementary material for: Novel Plastid Genome Characteristics in Fugacium kawagutii and the Trend of Accelerated Evolution of Plastid Proteins in Dinoflagellates
Source: Genome Biol Evol. 2023 Dec 29;16(1):evad237. doi: 10.1093/gbe/evad237 (PMC10781511; doi:10.1093/gbe/evad237)
Supplement: evad237_Supplementary_Data [file evad237_supplementary_data.zip › Supplementary Figures.pdf]

Supplementary Figures

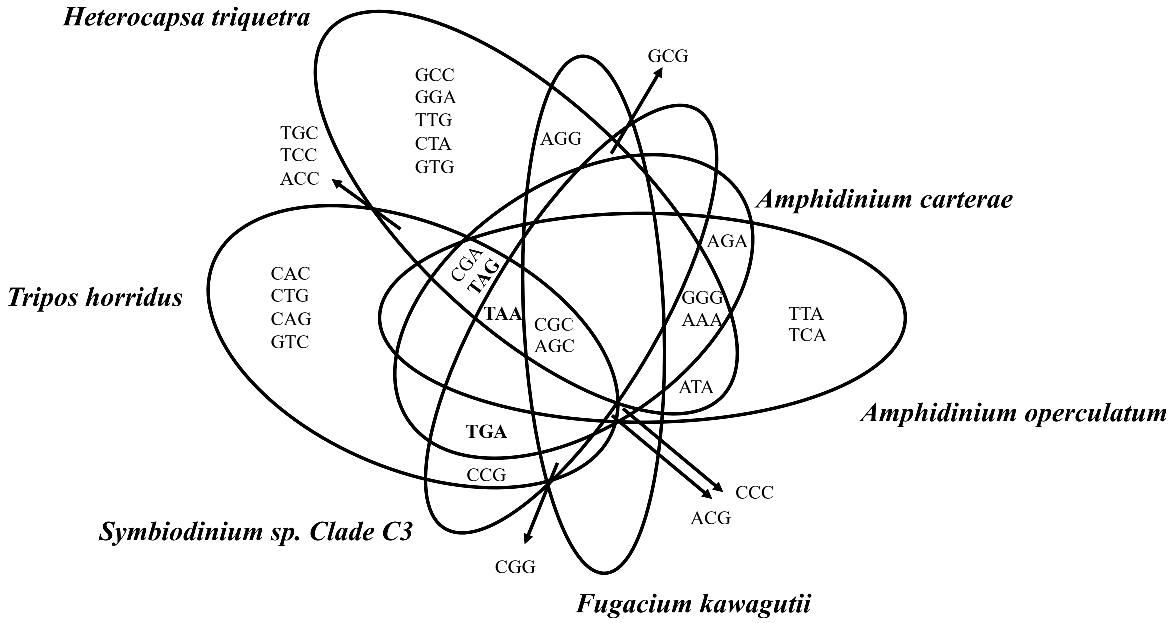

Figure S1. Venn Diagram showing the distribution of rare codons (which are used 10 or fewer times) in six dinoflagellate species. Stop codons are shown in bold.

A. Heterocapsaceae

1. *Heterocapsa arctica*

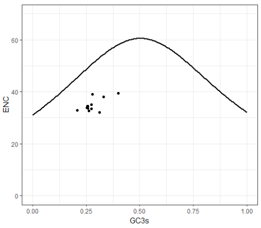

2. *Heterocapsa niei*

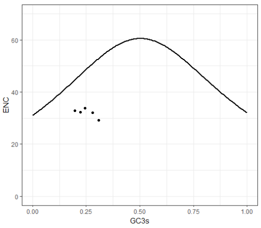

3. *Heterocapsa rotundata*

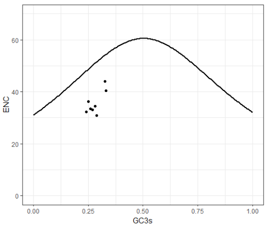

4. *Heterocapsa triquetra*

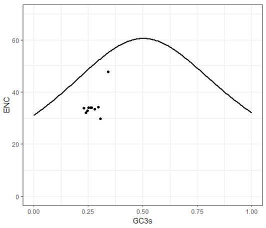

B. Amphidiniaceae

5. *Amphidinium carterae CCMP1314*

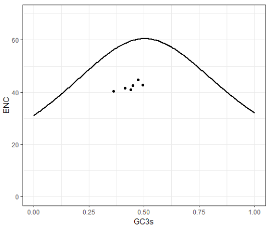

6. *Amphidinium massartii*

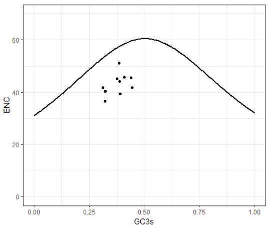

7. *Amphidinium operculatum*

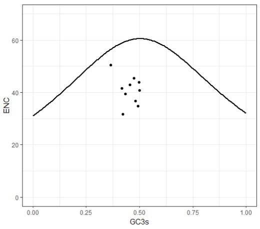

C. Prorocentraceae

8. *Prorocentrum CCMP2233*

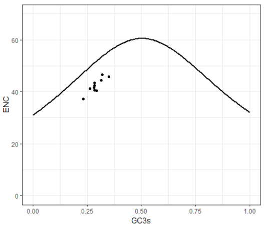

9. *Prorocentrum CCMP1329*

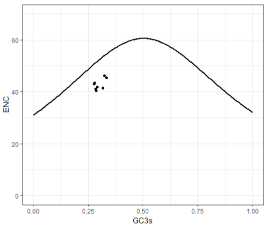

D. Pyrocystaceae

10. *Alexandrium andersonii*

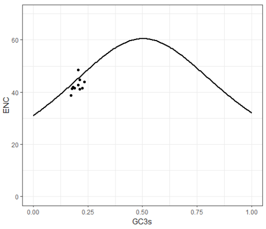

11. *Alexandrium catenella*

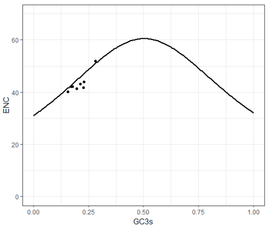

12. *Alexandrium margalefi*

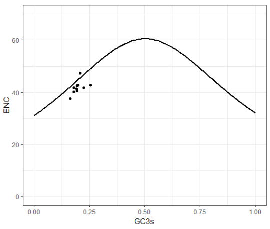

13. *Alexandrium minutum*

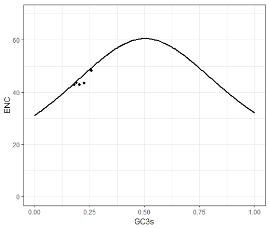

14. *Alexandrium monilatum*

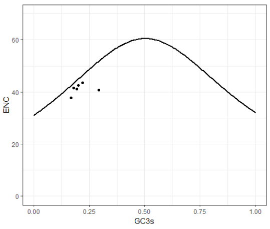

15. *Alexandrium ostenfeldii*

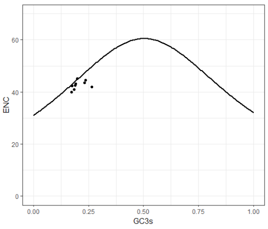

16. *Alexandrium tamarense*

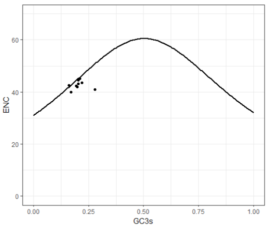

17. *Pyrodinium bahamense*

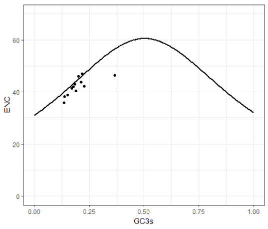

18. *Gambierdiscus australes*

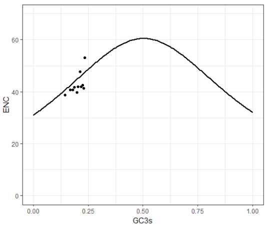

E. Ceratiaceae

19. *Ceratium fusus*

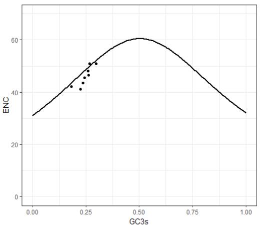

20. *Tripos horridus*

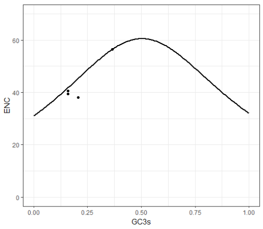

F. Lingulodiniaceae

21. *Lingulodinium polyedra*

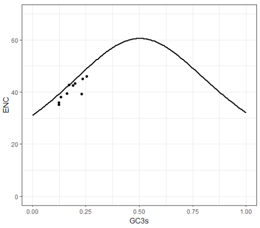

G. Gonyaulacaceae

22. *Gonyaulax spinifera*

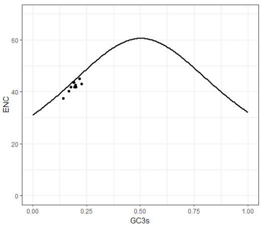

H. Protoceratiaceae

23. *Protoceratium reticulatum*

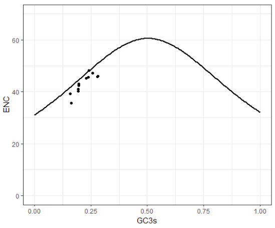

I. Amphidomataceae

24. *Azadinium spinosum*

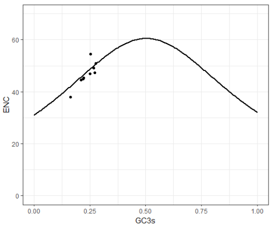

J. Symbiodiniaceae

25. *Symbiodinium C3* 26. *Symbiodinium C15* 27. *Symbiodinium CCMP421* 28. *Symbiodinium CCMP2430*

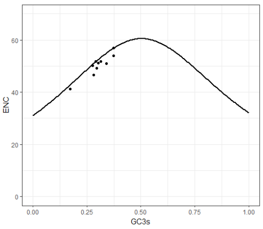

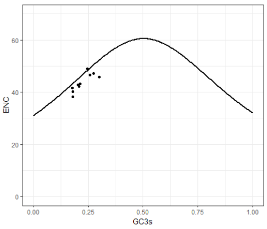

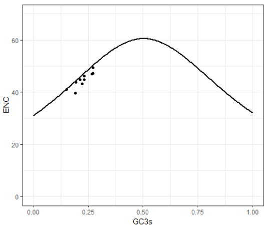

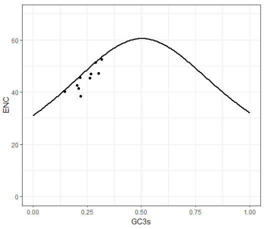

29. *Symbiodinium D1a* 30. *Symbiodinium Mf 1.05b* 31. *Symbiodinium Mp* 32. *Fugacium kawagutii*

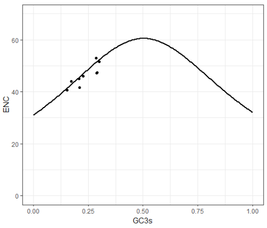

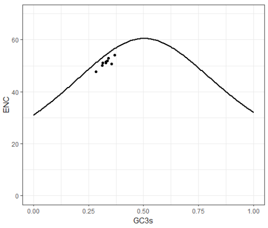

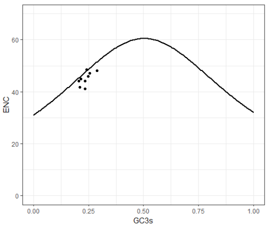

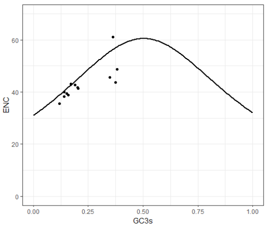

33. *Cladocopium goreaui*

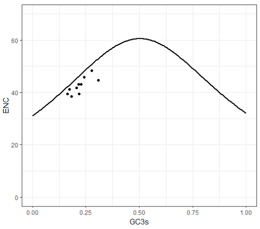

K. Thoracosphaeraceae

34. *Apocalathium aciculiferum*

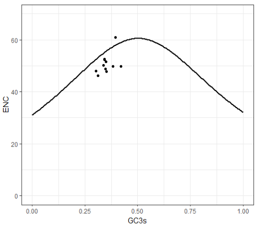

L. Peridiniaceae

35. *Scrippsiella SHHI4* 36. *Scrippsiella SHTV5* 37. *Scrippsiella acuminata* 38. *Brandtodinium nutriculum*

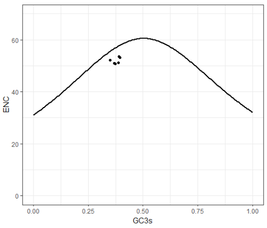

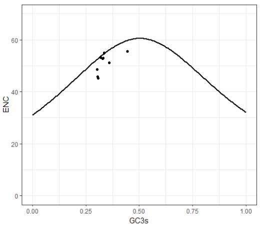

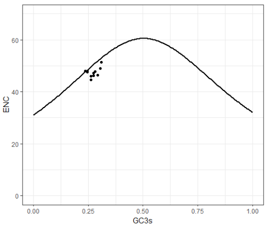

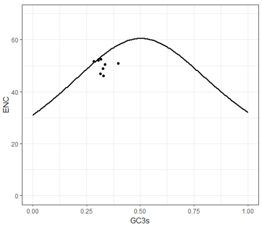

M. Suessiaceae

39. *Polarella glacialis* 40. *Pelagodinium beii*

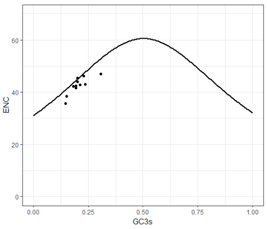

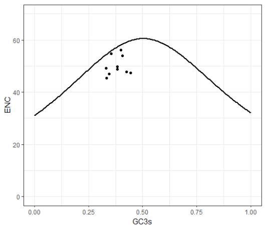

Figure S2. The specific ENc-plot analysis (ENc plotted against GC_{3S}). ENc denotes the effective number of codons, and GC_{3S} denotes the GC content on the third synonymous codon position. The expected ENc from GC_{3S} are shown as a standard curve. *Scrippsiella acuminata* was formerly *S.*

trochoides. A) The ENc-plot analysis of Heterocapsaceae. B) The ENc-plot analysis of Amphidiniaceae. C) The ENc-plot analysis of Prorocentraceae. D) The ENc-plot analysis of Pyrocystaceae. E) The ENc-plot analysis of Ceratiaceae. F) The ENc-plot analysis of Lingulodiniaceae. G) The ENc-plot analysis of Gonyaulacaceae. H) The ENc-plot analysis of Protoceratiaceae. I) The ENc-plot analysis of Amphidomataceae. J) The ENc-plot analysis of Symbiodiniaceae. K) The ENc-plot analysis of Thoracosphaeraceae. L) The ENc-plot analysis of Peridiniacea. M) The ENc-plot analysis of Suessiaceae.

A. Heterocapsaceae

1. *Heterocapsa arctica*

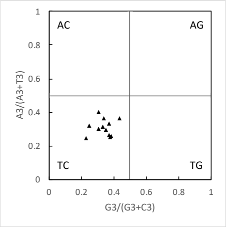

2. *Heterocapsa niei*

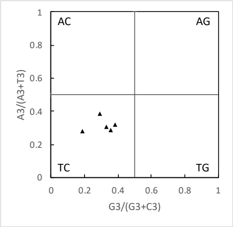

3. *Heterocapsa rotundata*

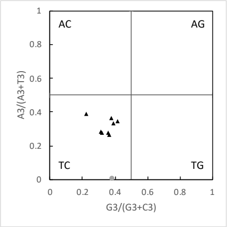

4. *Heterocapsa triquetra*

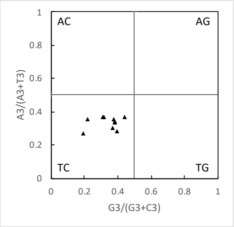

B. Amphidiniaceae

5. *Amphidinium carterae CCMP1314*

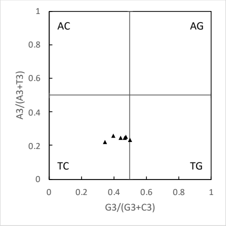

6. *Amphidinium massartii.*

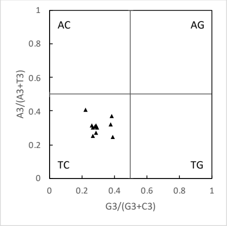

7. *Amphidinium operculatum*

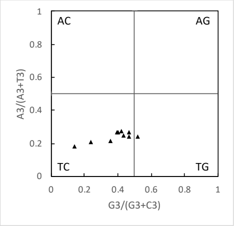

C. Prorocentraceae

8. *Prorocentrum CCMP2233*

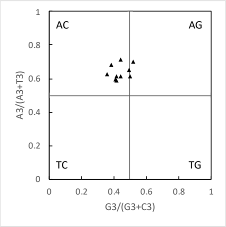

9. *Prorocentrum CCMP1329*

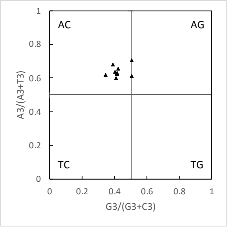

D. Pyrocystaceae

10. *Alexandrium andersonii*

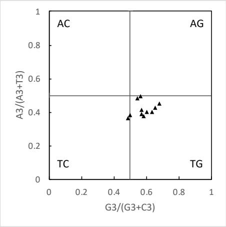

11. *Alexandrium catenella*

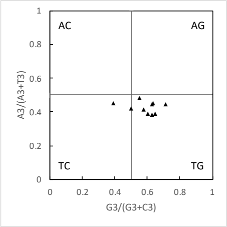

12. *Alexandrium margalefi*

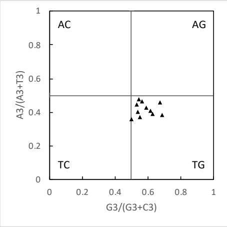

13. *Alexandrium minutum*

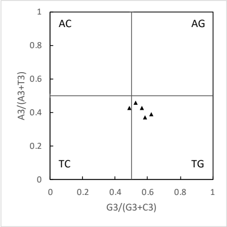

14. *Alexandrium monilatum*

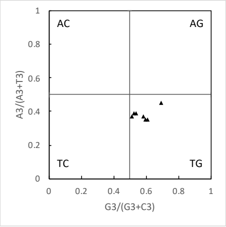

15. *Alexandrium ostenfeldii*

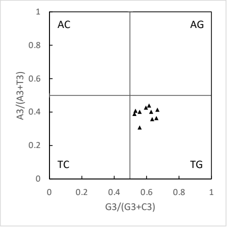

16. *Alexandrium tamarense*

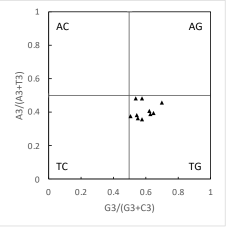

17. *Pyrodinium bahamense*

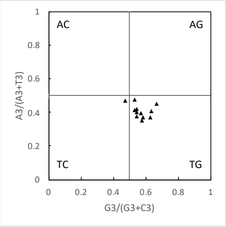

18. *Gambierdiscus australes*

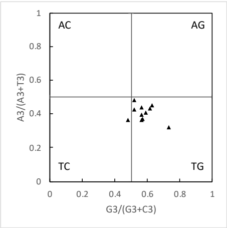

E. Ceratiaceae

19. *Ceratium fusus*

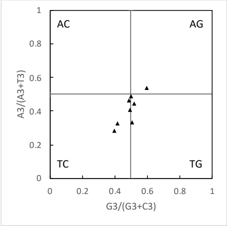

20. *Tripos horridus*

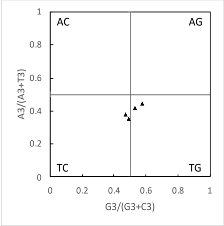

F. Lingulodiniaceae

21. *Lingulodinium polyedra*

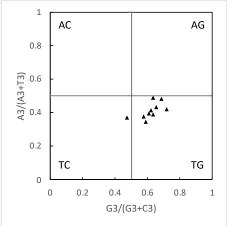

G. Gonyaulacaceae

22. *Gonyaulax spinifera*

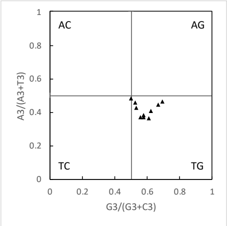

H. Protoceratiaceae

23. *Protoceratium reticulatum*

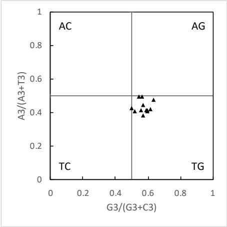

I. Amphidomataceae

24. *Azadinium spinosum*

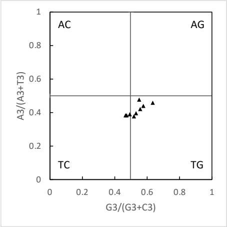

J. Symbiodiniaceae

25. *Symbiodinium C3*

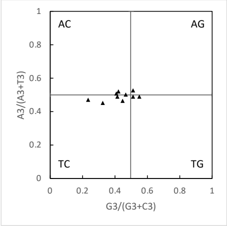

26. *Symbiodinium C15*

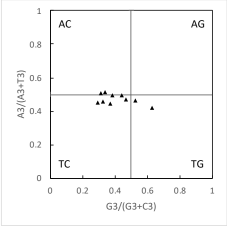

27. *Symbiodinium CCMP421*

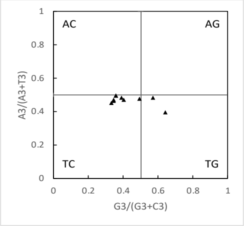

28. *Symbiodinium CCMP2430*

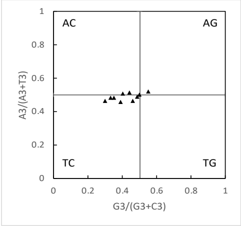

29. *Symbiodinium D1a*

30. *Symbiodinium Mf 1.05b*

31. *Symbiodinium Mp*

32. *Fugacium kawagutii*

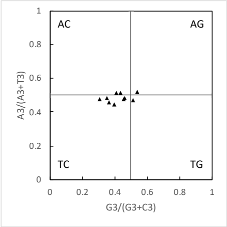

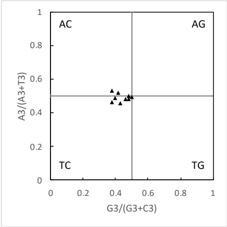

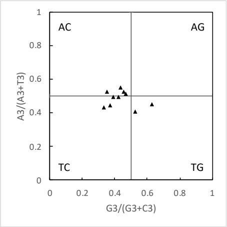

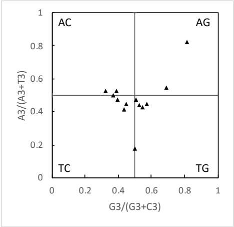

33. *Cladocopium goreaui*

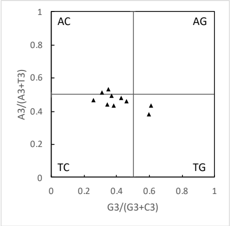

K. Thoracosphaeraceae

34. *Apocalathium aciculiferum*

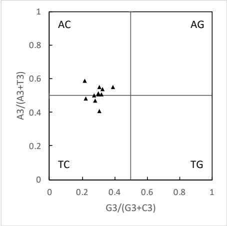

L. Peridiniaceae

35. *Scrippsiella SHHI4* 36. *Scrippsiella SHTV5* 37. *Scrippsiella acuminata* 38. *Brandtodinium nutriculum*

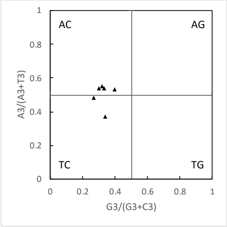

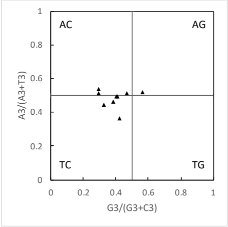

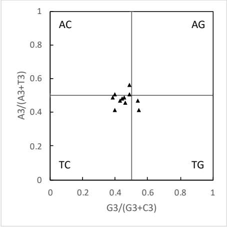

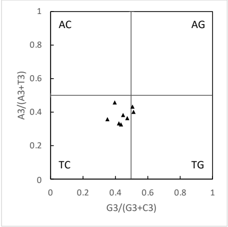

M. Suessiaceae

39. *Polarella glacialis* 40. *Pelagodinium beii*

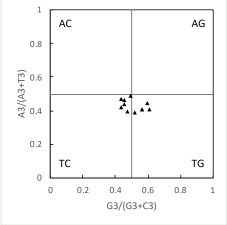

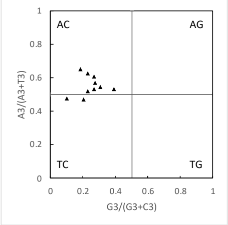

Figure S3. The specific Parity Rule 2 (PR2)-bias plot. [A_3 / (A_3 + T_3) against G_3 / (G_3 + C_3)]. A) The PR2-plot analysis of Heterocapsaceae. B) The PR2-plot analysis of Amphidiniaceae. C) The PR2-plot analysis of Prorocentraceae. D) The PR2-plot analysis of Pyrocystaceae. E) The PR2-plot analysis

of Ceratiaceae. F) The PR2-plot analysis of Lingulodiniaceae. G) The PR2-plot analysis of Gonyaulacaceae. H) The PR2-plot analysis of Protoceratiaceae. I) The PR2-plot analysis of Amphidomataceae. J) The PR2-plot analysis of Symbiodiniaceae. K) The PR2-plot analysis of Thoracosphaeraceae. L) The PR2-plot analysis of Peridiniacea. M) The PR2-plot analysis of Suessiaceae.

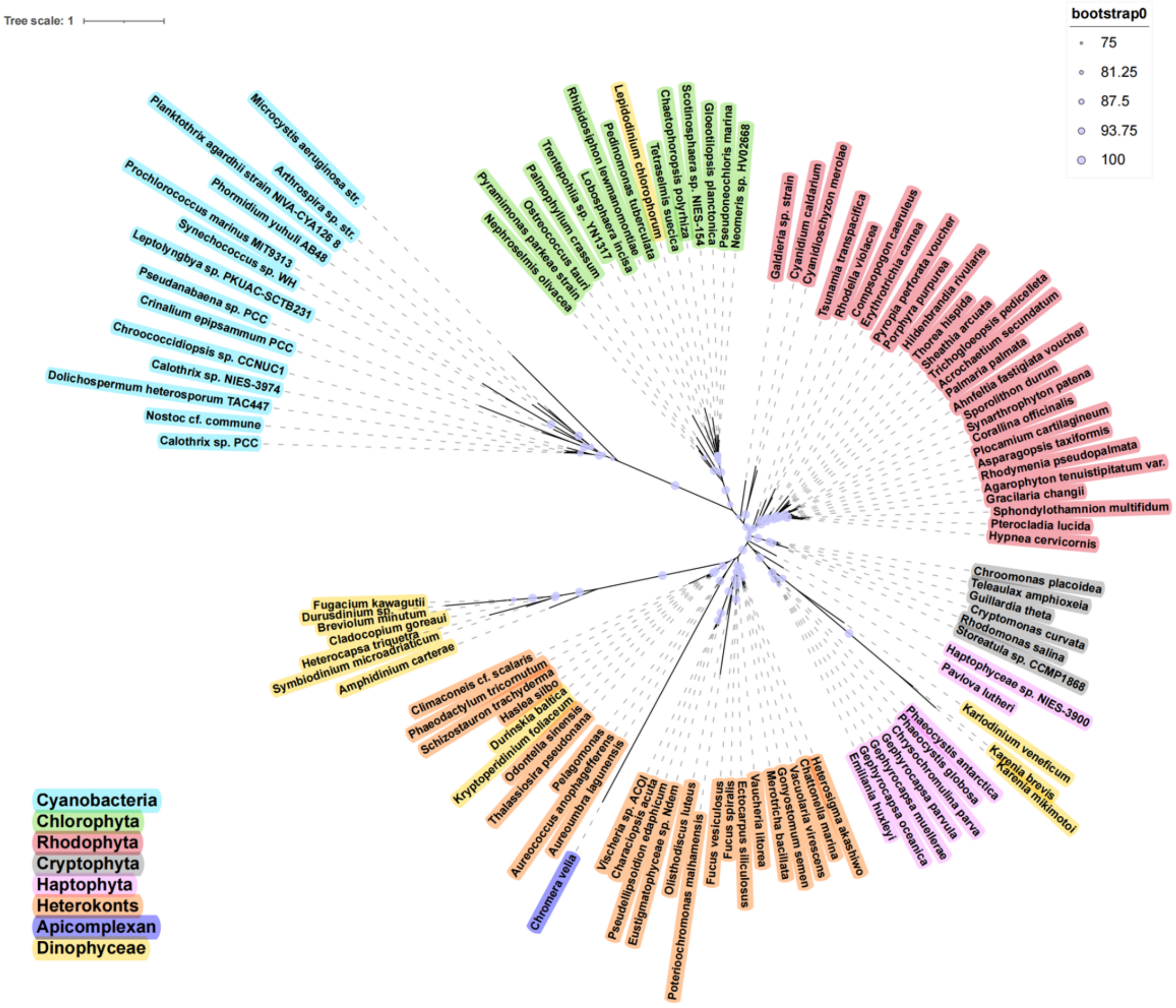

Figure S4. Multi-protein phylogeny of nucleus-encoded plastid proteins in peridinin dinoflagellate. Tree topology shown is from maximum-likelihood (ML) analysis for the five proteins of which encoding genes were transferred to the nucleus in peridinin dinoflagellate, but in other phyla/classes remain in the plastid. Circles at each node indicate support of nodes >75% in ML bootstrap values. Taxonomic groupings of dinoflagellates and other photosynthetic protists, apicomplexan and cyanobacteria are shown in different colors.

Alignment of *Cladocopium* core regions

Cladocopium proliferum SCF055	AT.GGGCTGGGTGCCCTACCCAGCCCATATGGGCCCACGCTTCC.GGGGCCCATAACGGCCCTTCGGGCCTTCAAAAA
Symbiodinium sp.C3 isolated from Agaricia sp	ATGGGGCTGGGTGCCCTACCCAGCCCATATGGGCCCACACTTCGTGGGGCCCATAACG.CCCTTCGGGC.TTCAAAAAATAAA
Symbiodinium sp. gt2	AT.GGGCTGGGTGCCCTACCCAGCCCATATGGGCCCACACTTCGTGGGGCCCATAACG.CCCTTCGGGC.TTCAAAAAATAAA
Symbiodinium sp. gt1	AT.GGGCTGGGTGCCCTACCCAGCCCATATGGGCCCACACTTCGTGGGGCCCATAACG.CCCTTCGGGC.TTCAAAAAATAAA
Cladocopium sp. clade C clone psb31	AT.GGGCTGGGTGCCCTACCCAGCCCATATGGGCCCACACTTCGTGGGGCCCATAACG.CCCTTCGGGC.TTCAAAAAATA..
Cladocopium sp. clade C clone psb30	AT.GGGCTGGGTGCCCTACCCAGCCCATATGGGCCCACACTTCGTGGGGCCCATAACG.CCCTTCGGGC.TTCAAAAAATA..
Cladocopium sp. clade C clone psb29	AT.GGGCTGGGTGCCCTACCCAGCCCATATGGGCCCACACTTCGTGGGGCCCATAACG.CCCTTCGGGC.TTCAAAAAATA..
Cladocopium sp. clade C clone psb25	AT.GGGCTGGGTGCCCTACCCAGCCCATATGGGCCCACACTTCGTGGGGCCCATAACG.CCCTTCGGGC.TTCAAAAAATA..
Symbiodinium sp. ex Scolymia sp	AT.GGGCTGGGTGCCCTACCCAGCCCATATGGGCCCACACTTCGTGGGGCCCATAACG.CCCTTCGGGC.TTCAAAAA.TAAA
Symbiodinium sp. DJT-2013A isolateRB 38	AT.GGGCTGGGTGCCCTACCCAGCCCATATGGGCCCACACTTCGTGGGGCCCATAACGGCCCTTCGGGCCTTCAAAAAATAA.
Symbiodinium sp. DJT-2013A isolateBar0584	AT.GGGCTGGGTGCCCTACCCAGCCCATATGGGCCCACACTTCGTGGGGCCCATAACGGCCCTTCGGGCCTTCAAAAAATAAA
Cladocopium sp. clade C clone psb118	AT.GGGCTGGGTGCCCTACCCAGCCCATATGGGCCCACACTTCGTGGGGCCCATAACG.CCCTTCGGGC.TTCAAAAA.....
Cladocopium sp. clade C clone psb113	AT.GGGCTGGGTGCCCTACCCAGCCCATATGGGCCCACACTTCGTGGGGCCCATAACG.CCCTTCGGGC.TTCAAAAA.....
Cladocopium sp. clade C clone psb110	AT.GGGCTGGGTGCCCTACCCAGCCCATATGGGCCCACACTTCGTGGGGCCCATAACG.CCCTTCGGGC.TTCAAAAA.....
Cladocopium sp. clade C clone psb28	AT.GGGCTGGGTGCCCTACCCAGCCCATATGGGCCCACACTTCGTGGGGCCCATAACG.CCCTTCGGGC.TTCAAAAA.....
Symbiodinium sp. C100 isolate T3PE03	AT.GGGCTGGGTGCCCTACCCAGCCCATATGGGCCCACGCTTCGCGGGGCCCATAACG.CCCTTCGGGC.TTCAAAA..TAAT
Symbiodinium sp. C100 isolate T3PE01	AT.GGGCTGGGTGCCCTACCCAGCCCATATGGGCCCACGCTTCGCGGGGCCCATAACG.CCCTTCGGGC.TTCAAAA..TAAT
Cladocopium sp. clade C clone psb60	ATGGGG.TGGGTGCCCTACCCACCCCATATGGGCCCACACTTCGTGGGGCCCATAACG.CCCTTCGGGC.TTCAAAAAATA..
Cladocopium sp. clade C clone psb7	ATGGGG.TGGGTGCCCTACCCACCCCATATGGGCCCACACTTCGTGGGGCCCATAACG.CCCTTCGGGC.TTCAAAAAATA..
Cladocopium sp. clade C clone psb6	ATGGGG.TGGGTGCCCTACCCACCCCATATGGGCCCACACTTCGTGGGGCCCATAACG.CCCTTCGGGC.TTCAAAAAATA..
Cladocopium sp. clade C clone psb4	ATGGGG.TGGGTGCCCTACCCACCCCATATGGGCCCACACTTCGTGGGGCCCATAACG.CCCTTCGGGC.TTCAAAAAATA..
Cladocopium sp. clade C clone psb2	ATGGGG.TGGGTGCCCTACCCACCCCATATGGGCCCACACTTCGTGGGGCCCATAACG.CCCTTCGGGC.TTCAAAAAATA..
Cladocopium sp. 2486705 isolate A07PttoKa	AT.GGGCTGGGTGCCCTACCCAGCCCATATGGGCCCACGCTTCGCGGGGCCCATAACGGCCCTTCGGGCCTTCAAAAAATAAA
Symbiodinium sp. BB07_307 C1bc	AT.GGGCTGGGTGCCCTACCCAGCCCATATGGGCCCACGCTTCGCGGGGCCCATAACGGCCCTTCGGGCCTTCAAAAAATAAA
Cladocopium sp. 2486705 isolateC29PytoKa	AT.GGGCTGGGTGCCCTACCCAGCCCATATGGGCCCACGCTTCGCGGGGCCCATAACGGCCCTTCGGGCCTTCAAAAAATAAA
Cladocopium sp. 2486705 isolateC27PytoKa	AT.GGGCTGGGTGCCCTACCCAGCCCATATGGGCCCACGCTTCGCGGGGCCCATAACGGCCCTTCGGGCCTTCAAAAAATAAA
Cladocopium sp. 2486705 isolateC22PytoKa	AT.GGGCTGGGTGCCCTACCCAGCCCATATGGGCCCACGCTTCGCGGGGCCCATAACGGCCCTTCGGGCCTTCAAAAAATAAA
Symbiodinium sp. C100 isolate T3PE11	...GGGCTGGGTGCCCTACCCAGCCCATATGGGCCCACGCTTCGCGGGGCCCATAACG.CCCTTCGGGC.TTCAAAA..TAAT
Symbiodinium sp. C100 isolate T3PE02	...GGGCTGGGTGCCCTACCCAGCCCATATGGGCCCACGCTTCGCGGGGCCCATAACG.CCCTTCGGGC.TTCAAAA..TAAT
Cladocopium sp. clade C clone psb130	AT.GGGCTGGGTGCCCTACCCAGCCCATATGGGCCCACGCTTCGCGGGGCCCATAACGGCCCTTCGGGCCTTCAAAAAATAAA
Cladocopium sp. clade C clone psb121	AT.GGGCTGGGTGCCCTACCCAGCCCATATGGGCCCACACTTCGTGGGGCCCATAACGGCCCTTCGGGCCTTCAAAAA.....
Cladocopium sp. clade C clone psb 102	..GGGG.TGGGTGCCCTACCCACCCCATATGGGCCCACACTTCGTGGGGCCCATAACG.CCCTTCGGGC.TTCAAAAAATA..
Cladocopium sp. clade C clone psb 66	..GGGG.TGGGTGCCCTACCCACCCCATATGGGCCCACACTTCGTGGGGCCCATAACG.CCCTTCGGGC.TTCAAAAAATA..
Cladocopium sp. clade C clone psb65	..GGGG.TGGGTGCCCTACCCACCCCATATGGGCCCACACTTCGTGGGGCCCATAACG.CCCTTCGGGC.TTCAAAAAATA..
Cladocopium sp. clade C clone psb62	..GGGG.TGGGTGCCCTACCCACCCCATATGGGCCCACACTTCGTGGGGCCCATAACG.CCCTTCGGGC.TTCAAAAAATA..
Cladocopium sp. clade C clone psb6	..GGGG.TGGGTGCCCTACCCACCCCATATGGGCCCACACTTCGTGGGGCCCATAACG.CCCTTCGGGC.TTCAAAAAATA..
Cladocopium latusorum isolate RF25	AT.GGGCTGGGTGCCCTACCCAGCCCATATGGGCCCACGCTTCGCGGGGCCCATAACGGCCCTTCGGGCCTTCAAAAAATAA.
Cladocopium latusorum isolate RF20	AT.GGGCTGGGTGCCCTACCCAGCCCATATGGGCCCACGCTTCGCGGGGCCCATAACGGCCCTTCGGGCCTTCAAAAAATAA.
Cladocopium latusorum isolate RF19	AT.GGGCTGGGTGCCCTACCCAGCCCATATGGGCCCACGCTTCGCGGGGCCCATAACGGCCCTTCGGGCCTTCAAAAAATAAA
Cladocopium latusorum isolate RF18	AT.GGGCTGGGTGCCCTACCCAGCCCATATGGGCCCACGCTTCGCGGGGCCCATAACGGCCCTTCGGGCCTTCAAAAAATAA.
Cladocopium latusorum isolate RF17	AT.GGGCTGGGTGCCCTACCCAGCCCATATGGGCCCACGCTTCGCGGGGCCCATAACGGCCCTTCGGGCCTTCAAAAAATAAA
Cladocopium latusorum isolate RF15	AT.GGGCTGGGTGCCCTACCCAGCCCATATGGGCCCACGCTTCGCGGGGCCCATAACGGCCCTTCGGGCCTTCAAAAAATAAA

**Cladocopium latusorum isolate RF13**	AT.GGGCTGGGTGCCCTACCCAGCCCATATGGGCCCACGCTTCGCGGGGCCCATAACGGCCCTTCGGGCCTTCAAAAAATAAA
**Cladocopium latusorum isolate RS15**	AT.GGGCTGGGTGCCCTACCCAGCCCATATGGGCCCACGCTTCGCGGGGCCCATAACGGCCCTTCGGGCCTTCAAAAAATAAA
**Cladocopium latusorum isolate RS14**	AT.GGGCTGGGTGCCCTACCCAGCCCATATGGGCCCACGCTTCGCGGGGCCCATAACGGCCCTTCGGGCCTTCAAAAAATAAA
**Cladocopium latusorum isolate RS12**	AT.GGGCTGGGTGCCCTACCCAGCCCATATGGGCCCACGCTTCGCGGGGCCCATAACGGCCCTTCGGGCCTTCAAAAAATAAA
**Cladocopium latusorum isolate RS08**	AT.GGGCTGGGTGCCCTACCCAGCCCATATGGGCCCACGCTTCGCGGGGCCCATAACGGCCCTTCGGGCCTTCAAAAAATAAA
**Cladocopium latusorum isolate RS06**	AT.GGGCTGGGTGCCCTACCCAGCCCATATGGGCCCACGCTTCGCGGGGCCCATAACGGCCCTTCGGGCCTTCAAAAAATAAA
**Cladocopium latusorum isolate RS03**	AT.GGGCTGGGTGCCCTACCCAGCCCATATGGGCCCACGCTTCGCGGGGCCCATAACGGCCCTTCGGGCCTTCAAAAAATAAA
**Cladocopium latusorum isolate RS01**	AT.GGGCTGGGTGCCCTACCCAGCCCATATGGGCCCACGCTTCGCGGGGCCCATAACGGCCCTTCGGGCCTTCAAAAAATAAA
**Symbiodinium sp. DJT-2013d isolate Zam03**	AT.GGGCTGGGTGCCCTACCCAGCCCATATGGGCCCACGCTTCGCGGGGCCCATAACGGCCCTTCGGGCCTTCAAAAAATAAA
**Symbiodinium sp.DJT-2013A isolate CVX09_01**	AT.GGGCTGGGTGCCCTACCCAGCCCATATGGGCCCACGCTTCGCGGGGCCCATAACGGCCCTTCGGGCCTTCAAAAAATAAA
**Cladocopium pacificum isolate GAl_12_ 1287**	AT.GGGCTGGGTGCCCTACCCAGCCCATATGGGCCCACGCTTCGCGGGGCCCATAACGGCCCTTCGGGCCTTCAAAAAATAAA
**Cladocopium pacificum isolate Haw09_118**	AT.GGGCTGGGTGCCCTACCCAGCCCATATGGGCCCACGCTTCGCGGGGCCCATAACGGCCCTTCGGGCCTTCAAAAAATAAA
**Cladocopium pacificum isolate Pan09_ 17**	AT.GGGCTGGGTGCCCTACCCAGCCCATATGGGCCCACGCTTCGCGGGGCCCATAACGGCCCTTCGGGCCTTCAAAAAATAAA
**Cladocopium pacificum isolate Pan09_ 25**	AT.GGGCTGGGTGCCCTACCCAGCCCATATGGGCCCACGCTTCGCGGGGCCCATAACGGCCCTTCGGGCCTTCAAAAAATAAA
**Cladocopium pacificum isolate Davies_Poc1**	AT.GGGCTGGGTGCCCTACCCAGCCCATATGGGCCCACGCTTCGCGGGGCCCATAACGGCCCTTCGGGCCTTCAAAAAATAAA
**Cladocopium pacificum isolate Pan09_ 13**	AT.GGGCTGGGTGCCCTACCCAGCCCATATGGGCCCACGCTTCGCGGGGCCCATAACGGCCCTTCGGGCCTTCAAAAAATAAA
**Cladocopium pacificum isolate Pan09_ 82**	AT.GGGCTGGGTGCCCTACCCAGCCCATATGGGCCCACGCTTCGCGGGGCCCATAACGGCCCTTCGGGCCTTCAAAAAATAAA
**Cladocopium pacificum isolate Newcal_ 6**	AT.GGGCTGGGTGCCCTACCCAGCCCATATGGGCCCACGCTTCGCGGGGCCCATAACGGCCCTTCGGGCCTTCAAAAAATAAA
**Cladocopium pacificum isolate PAl09_ 471**	AT.GGGCTGGGTGCCCTACCCAGCCCATATGGGCCCACGCTTCGCGGGGCCCATAACGGCCCTTCGGGCCTTCAAAAAATAAA
**Cladocopium pacificum isolate Haw09_ 122**	AT.GGGCTGGGTGCCCTACCCAGCCCATATGGGCCCACGCTTCGCGGGGCCCATAACGGCCCTTCGGGCCTTCAAAAAATAAA
**Cladocopium pacificum isolate Haw09_ 97**	AT.GGGCTGGGTGCCCTACCCAGCCCATATGGGCCCACGCTTCGCGGGGCCCATAACGGCCCTTCGGGCCTTCAAAAAATAAA
**Cladocopium pacificum isolate TW37**	AT.GGGCTGGGTGCCCTACCCAGCCCATATGGGCCCACGCTTCGCGGGGCCCATAACGGCCCTTCGGGCCTTCAAAAAATAAA
**Cladocopium pacificum isolate TW59**	AT.GGGCTGGGTGCCCTACCCAGCCCATATGGGCCCACGCTTCGCGGGGCCCATAACGGCCCTTCGGGCCTTCAAAAAATAAA
**Cladocopium pacificum isolate Newcal_55**	AT.GGGCTGGGTGCCCTACCCAGCCCATATGGGCCCACGCTTCGCGGGGCCCATAACGGCCCTTCGGGCCTTCAAAAAATAAA
**Cladocopium pacificum isolate SML07_62**	AT.GGGCTGGGTGCCCTACCCAGCCCATATGGGCCCACGCTTCGCGGGGCCCATAACGGCCCTTCGGGCCTTCAAAAAATAAA
**Cladocopium pacificum isolate Phuket07_238**	AT.GGGCTGGGTGCCCTACCCAGCCCATATGGGCCCACGCTTCGCGGGGCCCATAACGGCCCTTCGGGCCTTCAAAAAATAAA
**Cladocopium latusorum isolate SML07_ 6**	AT.GGGCTGGGTGCCCTACCCAGCCCATATGGGCCCACGCTTCGCGGGGCCCATAACGGCCCTTCGGGCCTTCAAAAAATAAA
**Cladocopium latusorum isolate Zanzibar07_ 287**	AT.GGGCTGGGTGCCCTACCCAGCCCATATGGGCCCACGCTTCGCGGGGCCCATAACGGCCCTTCGGGCCTTCAAAAAATAAA
**Cladocopium latusorum isolate Zanzibar07_18**	AT.GGGCTGGGTGCCCTACCCAGCCCATATGGGCCCACGCTTCGCGGGGCCCATAACGGCCCTTCGGGCCTTCAAAAAATAAA
**Cladocopium latusorum isolate Phoenix09_220**	AT.GGGCTGGGTGCCCTACCCAGCCCATATGGGCCCACGCTTCGCGGGGCCCATAACGGCCCTTCGGGCCTTCAAAAAATAAA
**Cladocopium latusorum isolate Pan09_ 64**	AT.GGGCTGGGTGCCCTACCCAGCCCATATGGGCCCACGCTTCGCGGGGCCCATAACGGCCCTTCGGGCCTTCAAAAAATAAA
**Cladocopium latusorum isolate Pan09_ 28**	AT.GGGCTGGGTGCCCTACCCAGCCCATATGGGCCCACGCTTCGCGGGGCCCATAACGGCCCTTCGGGCCTTCAAAAAATAAA
**Cladocopium latusorum isolate Pal09_620**	AT.GGGCTGGGTGCCCTACCCAGCCCATATGGGCCCACGCTTCGCGGGGCCCATAACGGCCCTTCGGGCCTTCAAAAAATAAA
**Cladocopium latusorum isolate Newcal_ 31a**	AT.GGGCTGGGTGCCCTACCCAGCCCATATGGGCCCACGCTTCGCGGGGCCCATAACGGCCCTTCGGGCCTTCAAAAAATAAA
**Cladocopium latusorum isolate MVP_ POC3308**	AT.GGGCTGGGTGCCCTACCCAGCCCATATGGGCCCACGCTTCGCGGGGCCCATAACGGCCCTTCGGGCCTTCAAAAAATAAA
**Cladocopium latusorum isolate Aust03_77**	AT.GGGCTGGGTGCCCTACCCAGCCCATATGGGCCCACGCTTCGCGGGGCCCATAACGGCCCTTCGGGCCTTCAAAAAATAAA
**Cladocopium latusorum isolate Aust03_41**	AT.GGGCTGGGTGCCCTACCCAGCCCATATGGGCCCACGCTTCGCGGGGCCCATAACGGCCCTTCGGGCCTTCAAAAAATAAA
**Cladocopium latusorum isolate Aust02_139**	AT.GGGCTGGGTGCCCTACCCAGCCCATATGGGCCCACGCTTCGCGGGGCCCATAACGGCCCTTCGGGCCTTCAAAAAATAAA
**Symbiodinium sp. Panama27_C1bc**	AT.GGGCTGGGTGCCCTACCCAGCCCATATGGGCCCACGCTTCGCGGGGCCCATAACGGCCCTTCGGGCCTTCAAAAAATAAA
**Symbiodinium sp. Panama15_C1bc**	AT.GGGCTGGGTGCCCTACCCAGCCCATATGGGCCCACGCTTCGCGGGGCCCATAACGGCCCTTCGGGCCTTCAAAAAATAAA
**Symbiodinium sp. Panama11_C1bc**	AT.GGGCTGGGTGCCCTACCCAGCCCATATGGGCCCACGCTTCGCGGGGCCCATAACGGCCCTTCGGGCCTTCAAAAAATAAA
**Symbiodinium sp. BB07_308_C1bc**	AT.GGGCTGGGTGCCCTACCCAGCCCATATGGGCCCACGCTTCGCGGGGCCCATAACGGCCCTTCGGGCCTTCAAAAAATAAA
**Symbiodinium sp. GAL1060_C1d**	AT.GGGCTGGGTGCCCTACCCAGCCCATATGGGCCCACGCTTCGCGGGGCCCATAACGGCCCTTCGGGCCTTCAAAAAATAAA

Symbiodinium sp. Panama25_ C1d	AT.GGGCTGGGTGCCCTACCCAGCCCATATGGGCCCACGCTTCGCGGGGCCCATAACGGCCCTTCGGGCCTTCAAAAAATAAA
Symbiodinium sp. Panama17_ C1d	AT.GGGCTGGGTGCCCTACCCAGCCCATATGGGCCCACGCTTCGCGGGGCCCATAACGGCCCTTCGGGCCTTCAAAAAATAAA
Symbiodinium sp. Panama8_C1d	AT.GGGCTGGGTGCCCTACCCAGCCCATATGGGCCCACGCTTCGCGGGGCCCATAACGGCCCTTCGGGCCTTCAAAAAATAAA
Symbiodinium sp. Panama13_C1d	AT.GGGCTGGGTGCCCTACCCAGCCCATATGGGCCCACGCTTCGCGGGGCCCATAACGGCCCTTCGGGCCTTCAAAAAATAAA
Symbiodinium sp. Panama16_C1d	AT.GGGCTGGGTGCCCTACCCAGCCCATATGGGCCCACGCTTCGCGGGGCCCATAACGGCCCTTCGGGCCTTCAAAAAATAAA

Alignment of *Durusdinium* core reginons

Symbiodinium sp. clade D clone psb141	CACTGCTTTGGAGAGGTTGGACAGGTCTAATTATTAATAAATTTATTTATTTTAGTTTAAAGCTGCTCCACCTCCAAC
Symbiodinium sp. clade D clone psb139	CACTGCTTTGGAGAGGTTGGACAGGTCTAATTATTAATAAATTTATTTATTTTAGTTTAAAGCTGCTCCACCTCCAAC
Symbiodinium sp. clade D clone psb137	CACTGCTTTGGAGAGGTTGGACAGGTCTAATTATTAATAAATTTATTTATTTTAGTTTAAAGCTGCTCCACCTCCAAC
Symbiodinium sp. clade D clone psb138	CACTGCTTTGGAGAGGTTGGACAGGTCTAATTATTAATAAATTTATTAATT...........................
Symbiodinium sp. D1a isolate WA910	CACTGCTTTGGAGAGGTTGGACAGGTCTAATTATTAATAAATTTA................AGCTGCTCCACCTCCAAC
Symbiodinium sp. D1a isolate culture Mf10_ 8	CACTGCTTTGGAGAGGTTGGACAGGTCTAATTATTAATAAATTTA................AGCTGCTCCACCTCCAAC

Alignment of *Amphidinium carterae* core regions

AcCCMP1314 psbA	.TCCGGTCATTTTGTTCCATCTCTACCCCAGTAGAGAAAAATCCAGGTCATATCATAGGAGATGGAACTGAGAGATC
AcCCMP0512 J29-core	........CAATCGTTTCCATAAAGACACCAGCTTCAAAAAATGCCGGTCAATCCATAGGAG.TGAGAAAAT.....
AcCS21 psbB	TCTGGTCAAACTAGTCAATTTGGGTGCGAGTTTGGAATCTCAGCTCGATTCTCATAGG...................

Figure S5. Alignment of *Cladocopium*, *Durusdinium* and *Amphidinium carterae* non-coding minicircle sequences. The four bases are marked with different colors.
