# Supplementary figures and images for: Novel Plastid Genome Characteristics in Fugacium kawagutii and the Trend of Accelerated Evolution of Plastid Proteins in Dinoflagellates
Source: Genome Biol Evol. 2023 Dec 29;16(1):evad237. doi: 10.1093/gbe/evad237 (PMC10781511; doi:10.1093/gbe/evad237)

Tree scale: 1

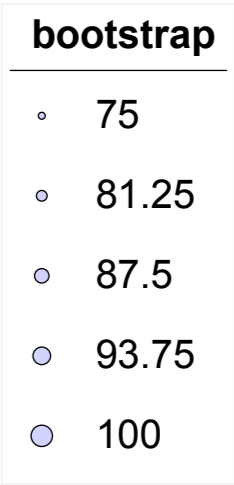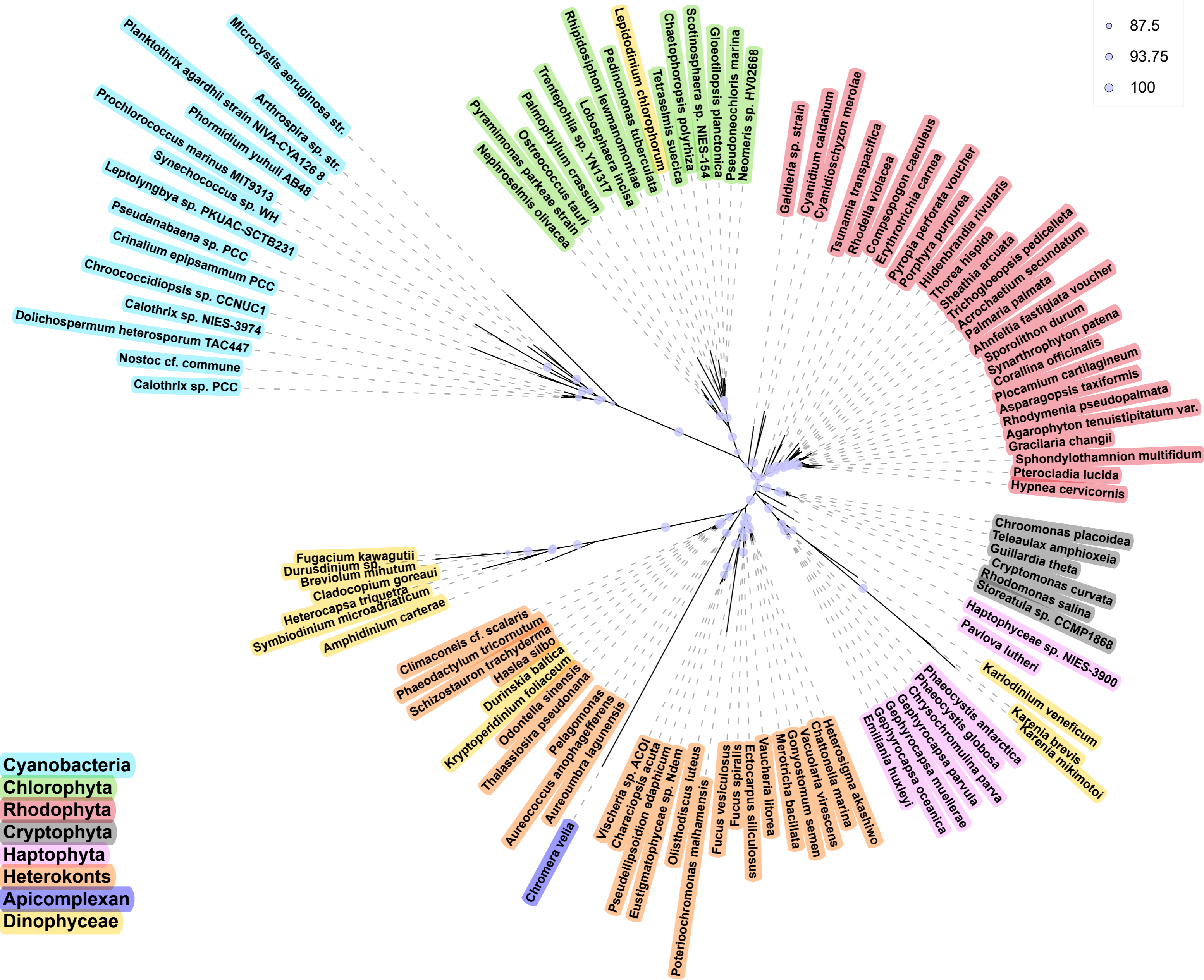

Supplement: evad237_Supplementary_Data [file evad237_supplementary_data.zip › Figure S4.pdf]
